# A spermidine riboswitch class in bacteria exploits a close variant of an aptamer for the enzyme cofactor *S*-adenosylmethionine

**DOI:** 10.1016/j.celrep.2023.113571

**Published:** 2023-12-12

**Authors:** Hubert Salvail, Aparaajita Balaji, Adam Roth, Ronald R. Breaker

**Affiliations:** 1 Department of Molecular, Cellular and Developmental Biology, Yale University, New Haven, CT 06520-8103, USA; 2 Howard Hughes Medical Institute, Yale University, New Haven, CT 06520-8103, USA; 3 Department of Molecular Biophysics and Biochemistry, Yale University, New Haven, CT 06520-8103, USA; 4 Lead contact

## Abstract

Natural polyamines such as spermidine and spermine cations have characteristics that make them highly likely to be sensed by riboswitches, such as their general affinity to polyanionic RNA and their broad contributions to cell physiology. Despite previous claims that polyamine riboswitches exist, evidence of their biological functions has remained unconvincing. Here, we report that rare variants of bacterial *S*-adenosyl-methionine-I (SAM-I) riboswitches reject SAM and have adapted to selectively sense spermidine. These spermidine-sensing riboswitch variants are associated with genes whose protein products are directly involved in the production of spermidine and other polyamines. Biochemical and genetic assays demonstrate that representatives of this riboswitch class robustly function as genetic “off” switches, wherein spermidine binding causes premature transcription termination to suppress the expression of polyamine biosynthetic genes. These findings confirm the existence of natural spermidine-sensing riboswitches in bacteria and expand the list of variant riboswitch classes that have adapted to bind different ligands.

## In brief

Salvail et al. demonstrate that rare variants of bacterial *S*-adenosylmethionine-I (SAM-I) riboswitches reject SAM and selectively sense spermidine to repress the expression of polyamine biosynthesis genes. This unusual class of RNA genetic switches expands the list of variant riboswitches that have evolved altered ligand-binding specificity.

## Graphical Abstract

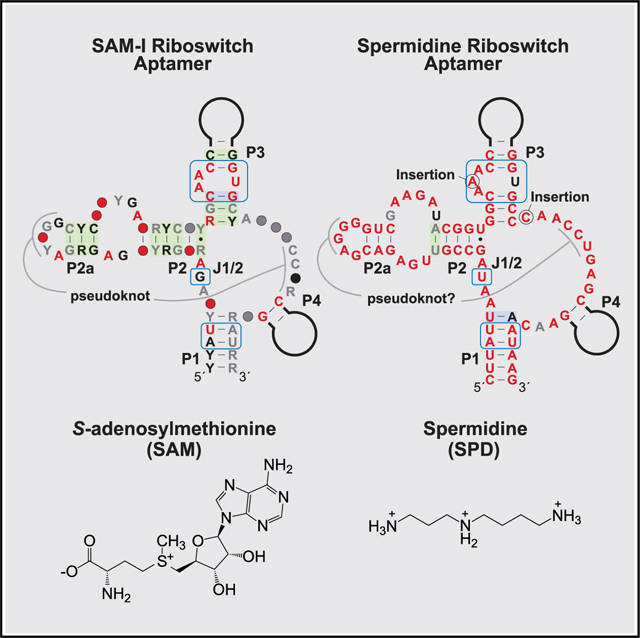

## INTRODUCTION

Riboswitches are structured RNAs located almost exclusively in the noncoding regions of mRNAs, where they selectively sense their target ligands and regulate gene expression.^1–4^ More than 55 distinct bacterial riboswitch classes have been reported to date,^4^ and the list of known ligands sensed by these RNAs^5^ includes various common metabolites, most enzyme cofactors and signaling molecules derived from RNA nucleotides or their precursors, and several elemental ions. In addition, riboswitch architectures have been found that exhibit cooperative ligand binding^6–10^ or that reside in tandem to generate more sophisticated gene control devices that yield steep dose-response curves,^6,11^ allosteric ribozyme function,^12^ or operate as two-input Boolean logic gates.^13^ These and other observations strongly indicate that RNA is a robust platform for the construction of molecular sensors and switches that are functionally competitive with their gene-control counterparts made of protein.

It has been proposed that many thousands of riboswitches likely remain to be discovered^1,4,14^ in the eubacterial domain of life. If true, it is then reasonable to assume that many other metabolites or other chemical entities are sensed by riboswitch classes that remain hidden in the genomes of bacteria. A productive strategy for the discovery of additional riboswitch classes involves the use of comparative sequence analysis algorithms to search for RNA motifs that exhibit characteristics of riboswitch function,^15–20^ such as conserved nucleotide sequences and structures residing in the noncoding portions of genomes. In addition, distinct riboswitch classes can be discovered by carefully evaluating putative members of a previously validated riboswitch class to identify sequence variants that associate with genes that have no relationship to the original ligand.^21,22^ When riboswitch variants are encountered,^21–31^ the overall architecture of the riboswitch aptamer is usually preserved, whereas the ligand binding core has mutated to recognize a different ligand.

Given the capabilities of riboswitches noted above, it seems certain that the list of metabolites proven to be sensed by RNA will greatly expand as efforts to discover additional riboswitches continue.^4^ This view has driven the longstanding speculation that all types of nucleotide-like enzyme cofactors and signaling molecules may eventually be matched with corresponding riboswitch classes.^4,32^ Indeed, although riboswitches for NAD^+^ had been notably missing, recent reports^33,34^ have revealed the existence of two distinct structural classes^35,36^ for this ubiquitous enzyme cofactor.

Similarly, compelling reasons support the hypothesis that polyamine-sensing riboswitches also likely exist. Spermidine and other polyamines are exceedingly abundant in cells,^37^ and they are notable for their strong interactions with nucleic acids.^37–39^ In *Escherichia coli*, approximately 90% of spermidine (estimated to be greater than 6 mM) is bound to RNA.^40,41^ Furthermore, polyamines have long been known to generally affect ribosome function during the process of translation.^38,39^ A common effect of molecules such as spermidine is to alter the speed of translation to influence the frequency of ribosome frameshifting, such that alternative proteins are made from the same mRNA.^42^

Because of these favorable biochemical properties, researchers have made numerous attempts (mostly unpublished) to discover and experimentally validate the existence of natural spermidine riboswitches. Most compelling would be the demonstration that spermidine directly and selectively binds a riboswitch aptamer located in an mRNA relevant to polyamine homeostasis or function. It has recently been claimed^43^ that the 5° UTR of the *spe2* mRNA from *Schizosaccharomyces pombe* carries a specialized aptamer for spermidine and that polyamine binding to this RNA regulates gene expression. However, we remain skeptical of the conclusions of this study due to the lack of strong evidence for riboswitch function and the reported varying gene expression trends in response to spermidine addition. Furthermore, it seems unlikely, as claimed, that a riboswitch would bind spermidine and increase expression of a gene whose protein product is an enzyme that participates in making more of this same polyamine.

Despite skepticism regarding recent claims and speculations on the existence of polyamine riboswitches, spermidine has previously been observed to bind mRNAs in a manner that could be important for regulating gene expression. For example, the effects on *oppA* gene expression in *E. coli* resulting from spermidine binding to a bulged region of RNA have been reported.^44,45^ Riboswitches typically have clear connections between the concentrations of the ligand sensed by the RNA genetic switch and the genes they regulate. Unfortunately, there is no clear connection between spermidine and the biological role of the OppA protein. Such a connection is also lacking for examples of T-box RNAs that have been shown to benefit from binding by spermidine.^46,47^ Thus, there have been no definitive demonstrations of spermidine riboswitch classes reported to date.

Here, we report that rare variants of the predominant *S*-adenosylmethionine-I (SAM-I) riboswitch class^48–50^ have adapted to selectively respond to spermidine. Although SAM-I riboswitches normally respond to the enzyme cofactor SAM, the rare variants reported in this study carry mutations at several nucleotide positions known to directly contact the SAM ligand,^51^ which presumably causes the variant riboswitches to reject SAM and selectively bind spermidine. Furthermore, genes associated with variant riboswitches are not always relevant to biological processes related to SAM or to sulfur homeostasis, which is common for SAM-I riboswitches.^48–50^ Rather, the variant riboswitches associate with several genes whose protein products are directly responsible for the biosynthesis of spermidine and other polyamines. Our findings demonstrate that some bacterial species likely have repurposed the architectural features of a SAM-I riboswitch to yield a distinct riboswitch class that naturally senses spermidine and regulates genes relevant to polyamine homeostasis.

## RESULTS AND DISCUSSION

### Identification of variants of the SAM-I riboswitch class

One approach to find more riboswitch classes is to search through the lists of representatives of known riboswitch classes to identify rare variants whose mutations and gene associations suggest that the RNAs have altered ligand specificities. This search strategy, either followed intentionally or otherwise, has yielded numerous additional riboswitch classes that sense ligands that are important to many species.^21–31^ We implemented this search strategy using RNAs that conform to the SAM-I riboswitch aptamer class, which is one of the most common ri boswitch classes in bacteria.^1,16^

**Figure 1.**
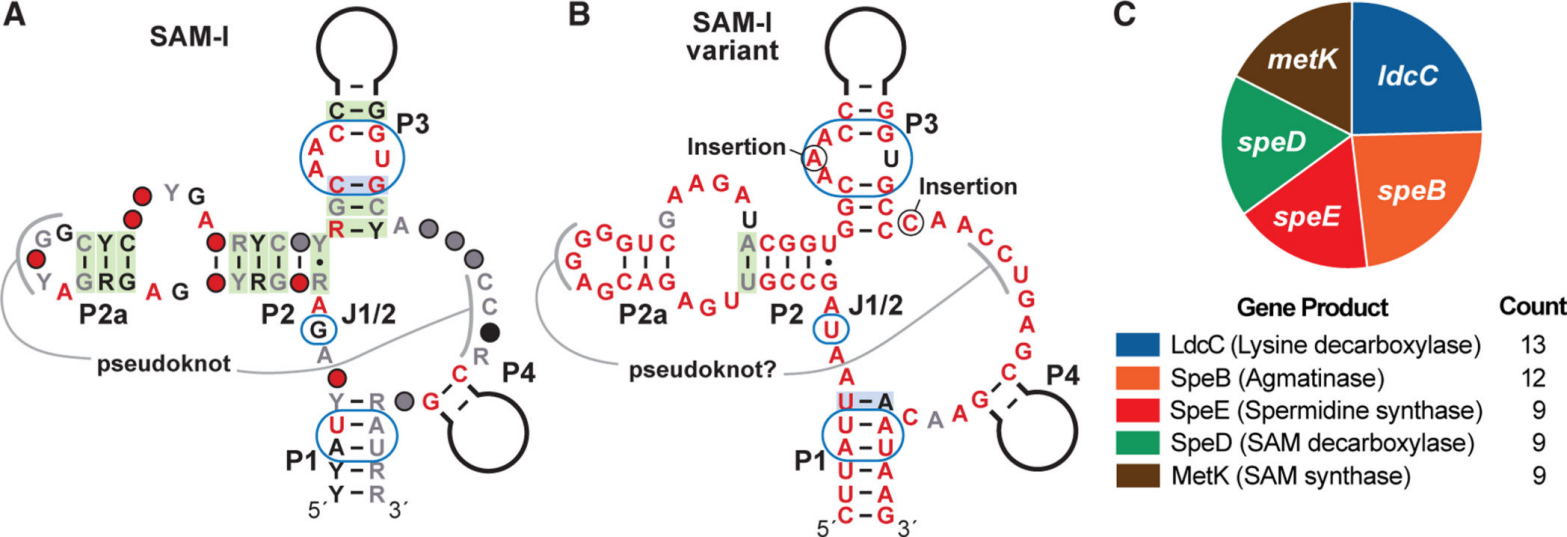
SAM-I and variant SAM-I riboswitch aptamers (A) Consensus sequence and secondary structure model for SAM-I riboswitch aptamers. Consensus model is based on that reported previously.^1^ P1–P4 identify 4 base-paired stems. J1/2 designates the joining region between stems P1 and P2. Red, black, and gray nucleotides are conserved in 97%, 90%, or 75% of the known representatives, respectively. Red, black, and gray circles indicate that nucleotides of any identity are present in 97%, 90%, or 75% of the known representatives, respectively. Thick black lines indicate variable-length sequences. Green shading indicates strong evidence of nucleotide covariation to maintain base pairing. Blue shading indicates strong evidence for compatible mutations that maintain base pairing. Blue ovals identify nucleotides that directly contact or reside immediately adjacent to the SAM ligand. (B) Consensus sequence and secondary structure model for SAM-I variants based on 13 distinct representatives identified using computational analysis (see Figure S1).^86,87^ Annotations are as described for (A). Note nucleotide changes in the J1/2 and P3 internal loop regions that alter nucleotides known to be important for SAM binding. (C) The distribution and abundances of genes associated with the SAM-I variant representatives.

Using a comparative sequence analysis approach, we searched a bacterial genomic DNA sequence dataset (RefSeq 96)^52^ to expand the number of known representatives that closely match the SAM-I riboswitch aptamer consensus (Figure 1A). By visually inspecting thousands of putative SAM-I riboswitch representatives, we identified 13 examples (Figure S1) of a variant aptamer (Figure 1B) that are unusual in part because they are found upstream of genes involved in the biosynthesis of the polyamines spermidine and cadaverine (Figure 1C). These hits are narrowly distributed across Gram-positive bacterial species from several genera, namely *Oceanobacillus*, *Bacillus*, *Trichococcus*, *Lysinibacillus*, *Viridibacillus*, *Sporosarcina*, and *Ornithinibacillus*, and from metagenomic DNA samples. These representatives are also unusual because they exhibit sequence differences in otherwise strongly conserved regions of SAM-I riboswitch aptamers that were previously reported^51^ to be involved in SAM recognition, thereby raising the possibility that they may have altered their ligand specificity.

### Evidence that SAM-I variants recognize a ligand related to polyamine biosynthesis

As part of the process of identifying the natural ligand for the SAM-I riboswitch variants, we considered the fact that SAM has many diverse roles in bacteria. For example, SAM is an essential methyl donor used by numerous methyltransferases, participates in radical SAM reactions, and serves as a precursor for the synthesis of other important metabolites.^53–56^ SAM also contributes to the production of bacterial signaling molecules involved in quorum sensing by serving as a substrate for the synthesis of *N*-homoserine lactone (autoinducer-1 [AI-1]) and as a precursor for the synthesis of AI-2.^56^ Thus, a wide range of candidate ligands for the variant RNAs was possible.

We also considered the fact that bacteria use several distinct classes of SAM-sensing riboswitches called SAM-I through SAM-VI and SAM-SAH,^57–60^ to closely monitor SAM concentrations. Some bacterial species use a riboswitch class selective for the SAM derivative called *S*-adenosylhomocysteine (SAH)^61^ to detect the accumulation of toxic amounts of this by-product of SAM-mediated methylation reactions.^62^ In addition, SAM-I riboswitches are used by many bacteria to regulate the expression of genes involved in SAM biosynthesis, but also evaluate SAM levels to regulate sulfur metabolism and the production of the sulfur-containing amino acids methionine and cysteine.^49^ Given that bacteria can use SAM levels as a surrogate to monitor the levels of other sulfur-containing metabolites, it is possible that some bacterial species may regulate genes tangentially related to SAM metabolism to control genes outside of the main pathway for SAM biosynthesis. Thus, close variants of SAM-I riboswitch aptamers may have adapted to sense one of several close derivatives of this ubiquitous coenzyme, or simply use SAM levels as a proxy to regulate a metabolic pathway that uses SAM. An example of the latter is the use of a SAM-I riboswitch that works in tandem with an AdoCbl riboswitch to regulate *metE* (MeCbl-independent methionine synthase) expression.^63^ These observations made us cautious in the pursuit of the function of the SAM-I variant riboswitches because they could represent a riboswitch subtype that simply recognizes SAM or a closely related ligand.

**Figure 2.**
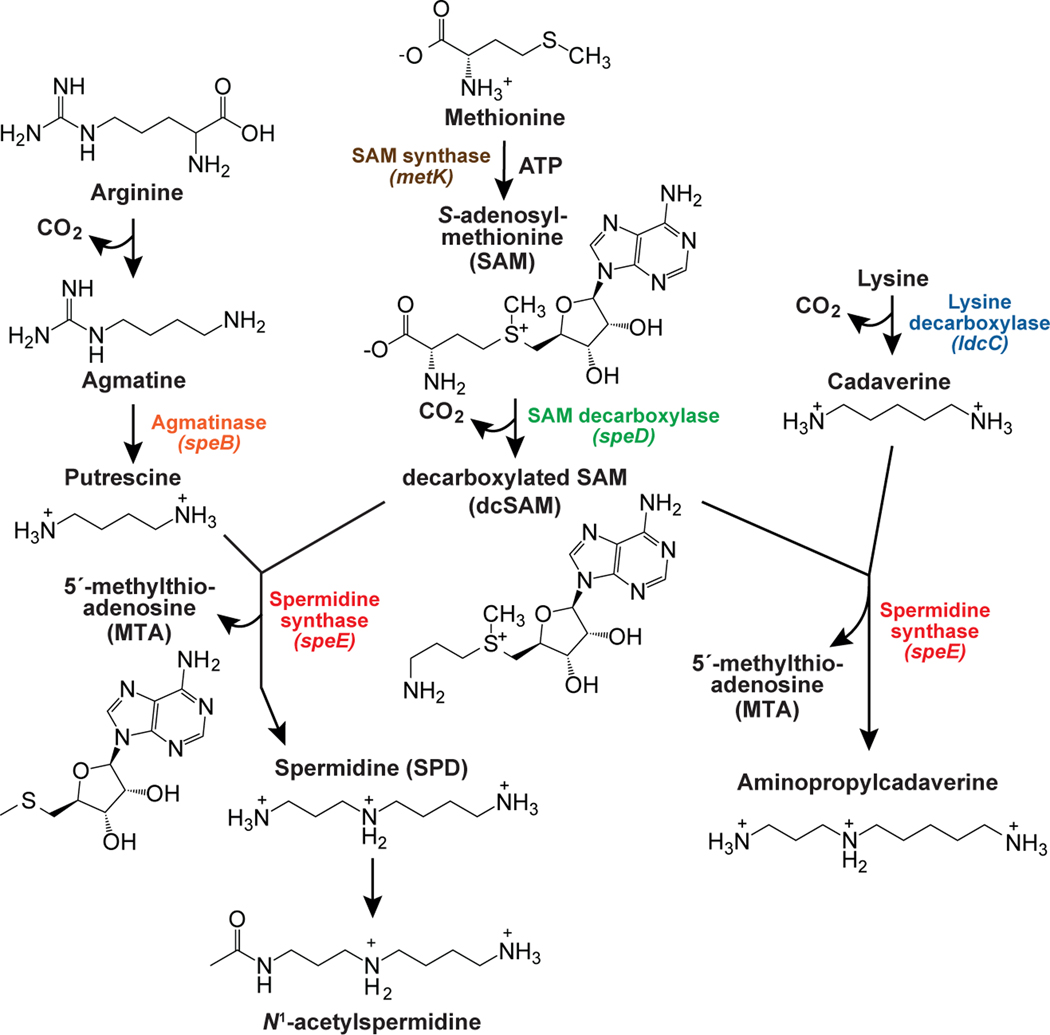
Polyamine biosynthesis pathways and the genes associated with variants of the SAM-I riboswitch class Bacterial polyamine biosynthesis. All of the genes/proteins identified (colored) are associated with variant riboswitches.

Despite the many possible ligand candidates to be considered for the variant class, we favored compounds that are directly relevant to spermidine due to the associations of the variant RNA with five genes relevant to polyamine biosynthesis (Figure 1C). Notably, SAM serves as a key substrate for the biosynthesis of polyamines in all three domains of life.^64^ In bacteria, spermidine is prepared by first converting SAM to decarboxylated SAM (dcSAM) (Figure 2). Through the action of spermidine synthase,^64,65^ an aminopropyl group is then transferred from dcSAM to putrescine to yield spermidine and the side product 5′-methylthioadenosine (MTA). Spermidine synthase also catalyzes the production of the polyamine aminopropylcadaverine from cadaverine and dcSAM in a reaction that generates MTA as a side product.^62^

Given the involvement of SAM in polyamine production (Figure 1C), we initially explored the possibility that the SAM-I riboswitch variants sensed either SAM, MTA, or some other SAM-like derivative that may serve as a proxy of the intracellular concentration of polyamines. SAM-I aptamers form a four-stem junction that positions conserved nucleotides from distal regions of the RNA to form a binding pocket that selectively recognizes SAM.^51^ Thus, it seemed mostlogical that the subtle RNA sequence changes present in the variant RNAs (Figure 1B) would not alter the global fold of each RNA. Rather, these changes were expected to permit the ligand binding pocket to recognize a ligand that was chemically similar to SAM. However, initial binding assays using SAM-like compounds failed to yield evidence for aptamer function. Therefore, we resorted to genetic assays to confirm riboswitch function before proceeding with additional binding assays.

### SAM-I riboswitch variants function as genetic “off” switches

**Figure 3.**
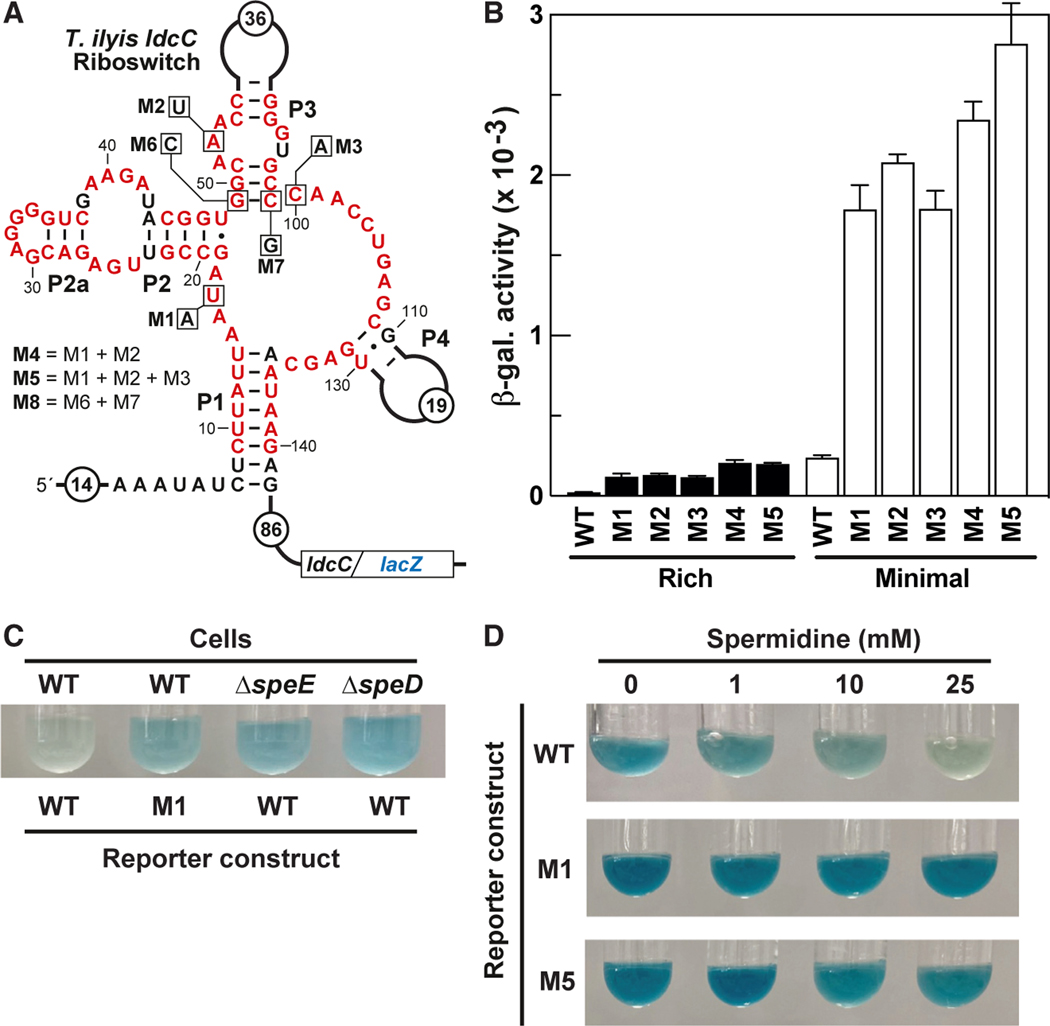
Spermidine represses gene expression regulated by a SAM-I variant riboswitch (A) Sequence and structural features of a SAM-I variant riboswitch associated with the *ldcC* gene from *T. ilyis* in a translational fusion with an *lacZ* reporter gene inserted into a pDG1661 vector.^88^ Red letters correspond to highly conserved nucleotides from the SAM-I variant consensus model depicted in Figure 1B. Boxed letters identify the nucleotides that were altered in mutant constructs M1–M8, which are evaluated as shown in (B) and Figures S3A–S3C. B) Reporter gene (β-galactosidase) expression of *B. subtilis* cells carrying either WT or mutant versions of the riboswitch-reporter fusion construct described in (A). Cells were grown in Luria-Bertani (LB) media or glucose minimal media (GMM) to late exponential phase, and reporter activity was then quantified by Miller assays.^89^ Error bars indicate the standard deviation for values derived from independent experiments (n = 3). (C) Effect of spermidine biosynthesis gene knockouts on reporter gene expression. WT, Δ*speD*, and Δ*speE B. subtilis* strains carrying the WT or M1 riboswitch-reporter fusion construct were grown for 18 h in GMM medium, and cultures were then supplemented with X-gal to visualize the reporter activity. Image is representative of 2 independent experiments, which gave similar results. D) Effect of spermidine supplementation on *ldcC-lacZ* gene reporter activity. *B. subtilis* cells carrying either the WT, M1, or M5 riboswitch-reporter fusion construct were grown to late exponential phase in GMM medium in the presence of the spermidine concentrations indicated. X-gal was then added to the cultures to visualize reporter activity. Images are representative of 2 independent experiments, which gave similar results.

To evaluate whether SAM-I variants retain gene regulation function, a genetic reporter construct was prepared by fusing the SAM-I variant representative from the *ldcC* gene of *Trichococcus ilyis* to a *lacZ* reporter gene. Transcription of this reporter system is driven by the *lysC* promoter from *Bacillus subtilis*, which is expected to constitutively express the engineered mRNA.^66^ This construct was integrated into the *amyE* locus of the surrogate organism *B. subtilis* (Figure 3A), and reporter activity was measured in liquid media cultures.

Cells harboring the wild-type (WT) riboswitch reporter exhibit substantially higher activity in minimal medium than in rich medium (Figure 3B). If the variant riboswitches are like most SAM-I riboswitches and turn off gene expression, then the ligand for this class is plentiful in cells grown in rich media and low in concentration when cells are grown in minimal media. Furthermore, reporter constructs carrying mutations in regions of the variant aptamer that correspond to the original SAM binding site (mutant constructs M1–M5) yield higher gene expression compared to the WT reporter construct (Figure 3B). Notably, the mutations do not completely disable ligand-dependent suppression of reporter gene activity, suggesting that ligand binding to the WT aptamer may be robust and is not easily disrupted by the mutations chosen for analysis. Regardless, these results are consistent with the hypothesis that variant riboswitches function as genetic off switches wherein ligand binding reduces gene expression.

### SAM-I riboswitch variants regulate gene expression in response to spermidine

Many of the representative SAM-I variants associate with genes coding for proteins involved in the biosynthesis of spermidine (i.e., *speE*, encoding spermidine synthase) or its precursors putrescine (i.e., *speB*, encoding agmatinase) and dcSAM (i.e., *speD*, encoding SAM decarboxylase) (Figures 1C and 2). Thus, we considered the possibility that spermidine, another similar polyamine, or a biosynthetic precursor may be the ligand for the variant riboswitch class. Given the fact that the variant RNAs appear to be genetic off switches, we favored the hypothesis that an end product of polyamine biosynthesis (e.g., spermidine) is the ligand. However, because of the similarity to SAM-I riboswitches in sequence and predicted structure, we also considered the possibility that the variant riboswitches turned off expression if MTA accumulated.

To test these hypotheses, the activity of the *T. ilyis ldcC* riboswitch reporter (Figure 3A) was examined in *B. subtilis* strains carrying genomic mutations that impair the production of MTA and spermidine (Δ*speE*), or of their precursor dcSAM (Δ*speD*) (Figure 2). When grown in minimal medium, WT riboswitch-reporter constructs exhibited higher gene expression in the Δ*speE* and Δ*speD B. subtilis* strains compared to WT cells (Figure 3C). The level of reporter expression in cells carrying a disrupted spermidine biosynthesis pathway was similar to that observed when the M1 riboswitch construct is used in WT cells grown in minimal medium. These results are consistent with the hypothesis that the SAM-I variant RNAs repress gene expression in response to spermidine binding or perhaps to MTA binding.

An important concern when conducting genetic reporter assays is the fact that spermidine may affect gene expression through a mechanism that does not involve the riboswitch under evaluation. As noted above, spermidine can affect other processes relevant to gene expression,^38,39,42^ and therefore we sought additional evidence that the SAM-I variant riboswitch from the *ldcC* gene of *T. ilyis* was mediating the observed changes in gene expression when spermidine biosynthesis was disrupted. This was achieved by conducting similar gene expression assays using a reporter construct wherein a consensus SAM-I riboswitch also from *T. ilyis* was fused to the *lacZ* gene (Figure S2A). This reporter construct, which is expected to turn off gene expression when SAM binds,^48–50^ yields low expression in WT *B. subtilis* cells, as well as in Δ*speE* and Δ*speD* cells in which spermidine biosynthesis is disrupted (Figure S2B). These results indicate that the sequence of the SAM-I variant riboswitch indeed is necessary for the observed boost in gene expression when cells are deficient in spermidine production.

Most striking, the addition of increasing spermidine concentrations to liquid media progressively decreases reporter expression mediated by the natural SAM-I variant riboswitch, but not that of the M1 and M5 constructs (Figure 3D). Recall that the M1 and M5 constructs carry mutations in the variant aptamer domain (Figure 3A) at nucleotide positions that differ between the variant class and the more common SAM-I aptamer class (Figure 1). These results strongly indicate that the natural variant riboswitch aptamer sequence is important for gene regulation and that spermidine (and not MTA) is the ligand for this rare class. Taken together, these results demonstrate that the SAM-I variant functions as a genetic off switch that suppresses the expression of polyamine biosynthesis genes in response to an increase in intracellular spermidine concentration.

### SAM-I variants modulate transcription termination in response to spermidine binding

**Figure 4.**
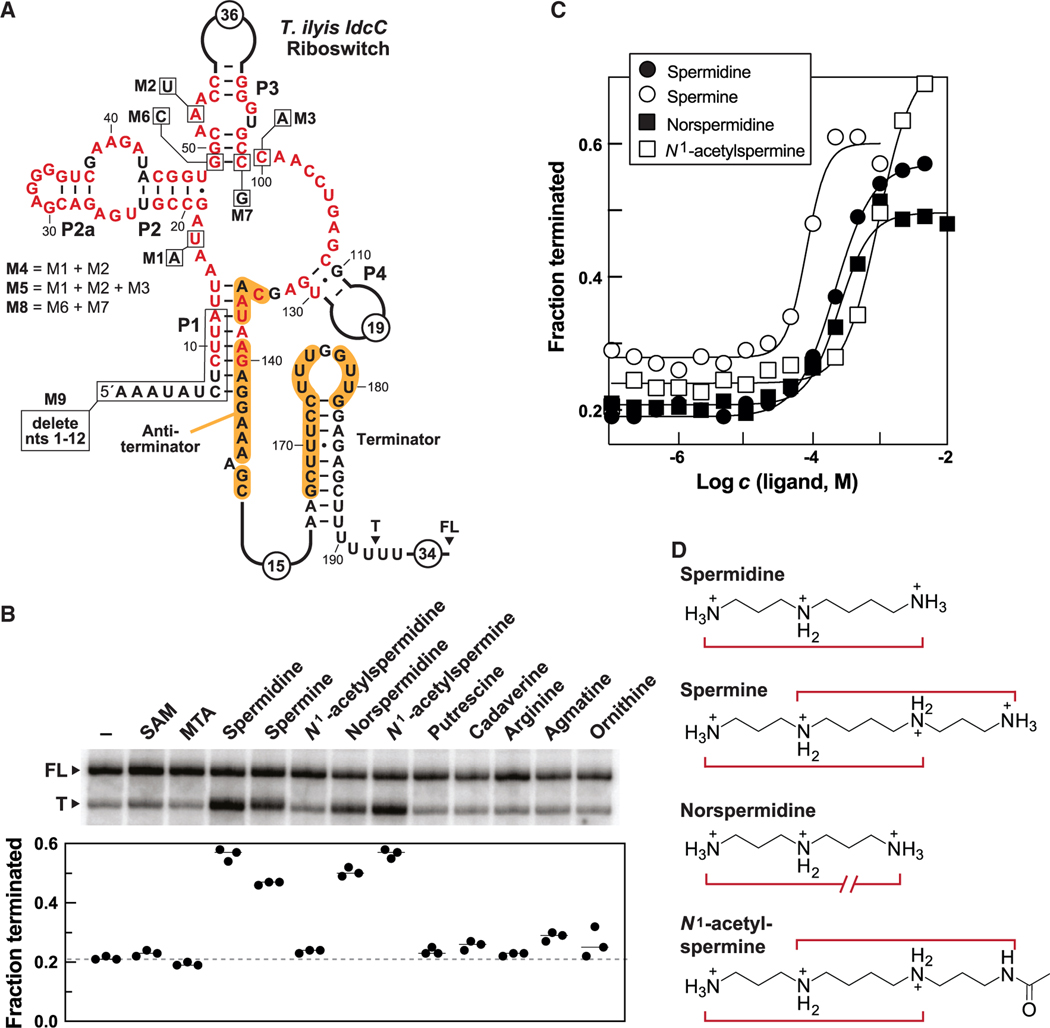
Transcription of a SAM-I variant riboswitch is terminated by spermidine and similar polyamines (A) Sequence and secondary structure model of a SAM-I variant riboswitch associated with the *ldcC* gene from *T. ilyis*. Approximate terminated (T) and FL 3′ ends are indicated. Other annotations are as described for Figure 3A. (B) Top: PAGE analysis of representative single-round transcription termination assays conducted in the absence (−) of a ligand candidate, or in the presence of 1 mM of the indicated compounds. Bottom: Values for the fraction of terminated RNA transcripts relative to the total amount of terminated and FL transcripts derived from independent experiments (n = 3). See also Figures S2C–S2F, S3, and S4 for transcription termination assays with mutant spermidine riboswitch constructs, other candidate ligands, or a SAM riboswitch. (C) Plot of the fraction of terminated *T. ilyis ldcC* riboswitch transcripts versus the logarithm of the molar concentration (*c*) of the ligand generated from the data presented in Figure S4. Plots were used to estimate the concentration of ligand required to cause half-maximal change in the fraction of terminated RNA (T_50_). (D) Chemical structures of polyamine compounds that trigger riboswitch-mediated transcription termination. Red enclosures identify regions of the compounds that are predicted to be recognized by the riboswitch.

Many SAM-I riboswitches are known^48–50,67^ to operate as transcriptional off switches by regulating the formation of an intrinsic terminator stem (a strong hairpin followed by a run of U nucleotides).^68,69^ Riboswitches commonly use an expression platform^1,4^ that exploits the mutually exclusive formation of terminator and antiterminator stems to regulate transcription in response to ligand binding. The *ldcC* variant SAM-I riboswitch from *T. ilyis* also carries a putative terminator stem whose left shoulder is predicted to be occluded via formation of an alternative antiterminator structure (Figure 4A). According to this model for the expression platform, ligand binding by the variant SAM-I riboswitch should prevent formation of the antiterminator stem, thereby permitting formation of the terminator and causing transcription to cease before the coding region of the mRNA is reached.

To determine whether spermidine directly terminates transcription mediated by a variant SAM-I riboswitch, we performed single-round transcription termination assays^70^ using a DNA template encompassing the *ldcC* variant SAM-I riboswitch from *T. ilyis* (Figure 4A). The expected full-length (FL) RNA transcript includes the aptamer, expression platform, and an additional 34 nucleotides residing 3′ of the poly-U region characteristic of intrinsic terminator stems.^68,69^ If the intrinsic terminator stem forms, then a terminated (T) RNA transcript ending within the poly-U region is expected to be produced.

In the absence of ligand, FL RNAs (~80%) predominate the distribution of transcripts produced from *in vitro* transcription reactions (Figure 4B). Furthermore, the addition of SAM, MTA, and some compounds analogous to polyamines fail to increase the fraction of early terminated transcripts (T). Although dcSAM was not commercially available, we tested decarboxylated SAH (dcSAH) as a close analog of dcSAM and observed no effect on transcription. These results exclude the possibility of bacteria using the variant riboswitch to sense SAM, the SAM-derived molecule MTA, and probably also dcSAM as a proxy for the concentration of spermidine. However, spermidine, spermine, norspermidine, and *N*^1^-acetylspermine cause a substantial increase in terminated transcripts, whereas various other amine-containing molecules do not affect product distribution (Figure 4B). Furthermore, mutant versions of the *T. ilyis ldcC* riboswitch construct (M1–M5) (Figure 4A) either diminish or eliminate spermidine-triggered transcription termination (Figure S3A). These results and those for related assays (Figures S3B and S3C) indicate that the SAM-I variant riboswitch has adapted to selectively sense spermidine and some of its close analogs.

Notably, the four active polyamine compounds require a similar concentration to half-maximally modulate transcription termination (T_50_) (Figures 4C and S4), suggesting that they share a common chemical structure that triggers riboswitch function. Each riboswitch-active ligand carries a 1,3-diaminopropyl group linked to the remainder of the molecule by a butyl chain (except for norspermidine) (Figure 4D). Spermine, which is a symmetrical molecule that could carry two (albeit overlapping) ligand substructures, also appears to be ~2-fold more potent (T_50_ = 79 μM) for inducing transcription termination than the other ligands such as spermidine (T_50_ = 214 μM) in initial assays (Figure 4C). Perhaps the symmetry of spermine increases the effective concentration of the ligand substructure by ~2-fold over that for spermidine. This hypothesis is consistent with the fact that *N*^1^-acetylspermidine fails to modulate transcription termination whereas *N*^1^-acetylspermine retains activity, likely due to the presence of a second ligand substructure. Intriguingly, the dose-response curves for transcription termination with these four ligands are steeper than expected for 1:1 RNA-ligand interactions, and this unusual characteristic is discussed further in the next section.

**Figure 5.**
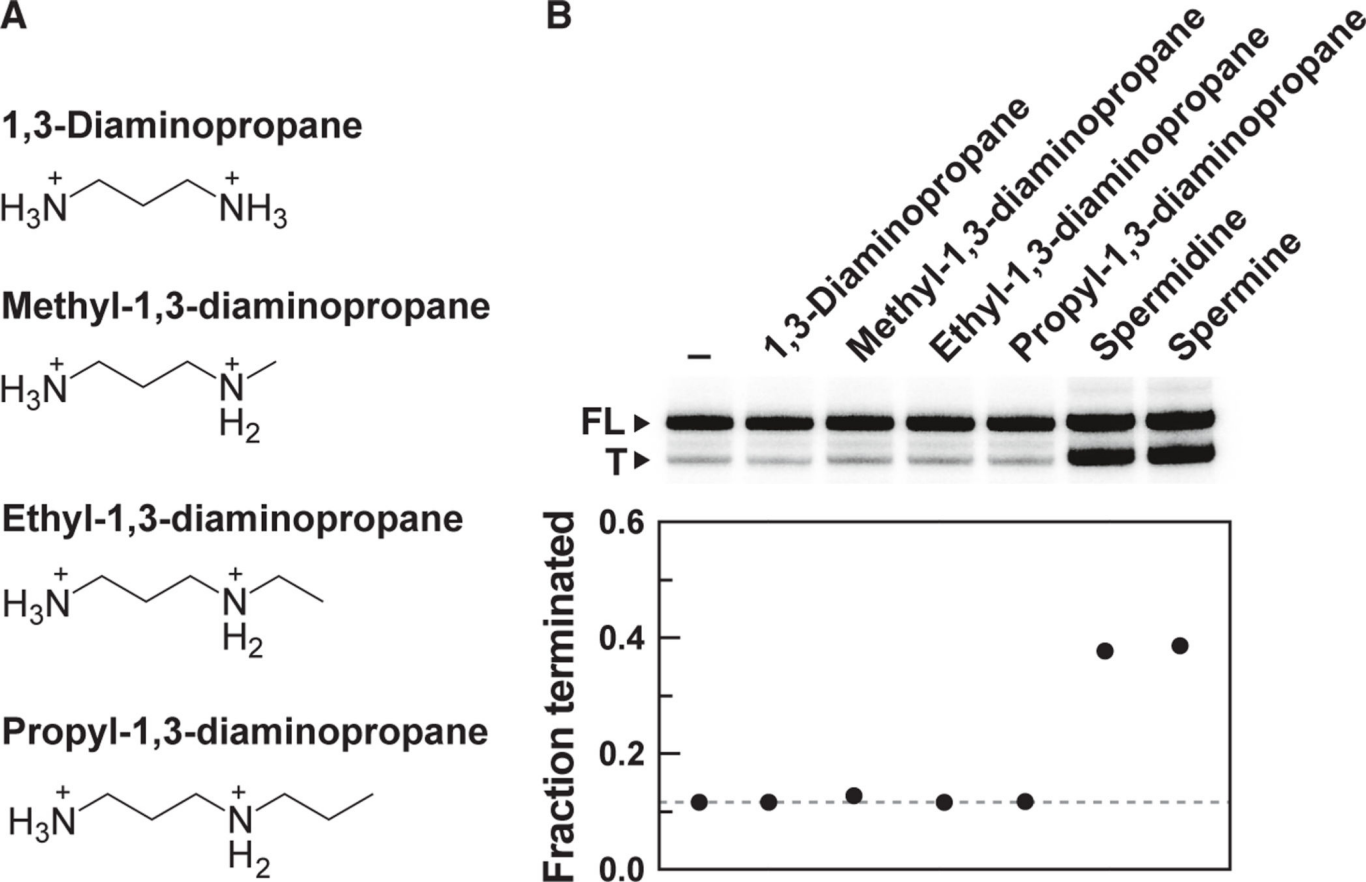
1,3-Diaminopropane and certain alkyl derivatives fail to trigger riboswitch-mediated transcription termination (A) Chemical structures of 1,3-diaminopropane and three alkyl derivatives representing fragments of spermidine. (B) Top: PAGE analysis of transcription termination assays conducted with *T. ilyis ldcC* SAM-I variant construct (Figure 4A) in the absence (−) or in the presence of 1 mM of the indicated compounds. Bottom: Plot of the values for the fraction of terminated RNA transcripts relative to the total amount of terminated and FL transcripts. Annotations are as described for Figure 4.

To further evaluate the chemical structure required for ligand function, additional transcription termination assays were conducted with 1,3-diaminopropane and its methyl, ethyl, and propyl derivatives (Figure 5A). These assays revealed that 1,3-diaminopropane and all of the tested alkyl derivatives fail to induce transcription termination (Figure 5B), indicating that these compounds lack the necessary molecular recognition determinants to affect transcription regulation. These findings suggest that all three amine groups of spermidine, and the analogous structures in the other active compounds, are likely recognized by the SAM-I variant. Presumably there is sufficient adaptability in the structure of the aptamer or of the ligand such that norspermidine can trigger riboswitch regulation, even though this compound is missing one methylene group between two of the amines compared to the other ligands (Figure 4D).

Among the various polyamines demonstrating effects on transcription termination mediated by the SAM-I variant (Figures 4 and 5), only spermidine appears to be physiologically relevant in the bacterial species carrying representatives of this riboswitch class. Furthermore, spermidine is the only polyamine produced by *B. subtilis*, the surrogate organism used in this study to demonstrate gene regulation by SAM-I variant riboswitch constructs (Figure 3). In contrast, other active polyamines such as spermine and *N*^1^-acetylspermine are not commonly found in bacteria.^71^ Norspermidine is only known to be synthesized by few bacterial species, such as *Vibrio cholerae*,^72^ that do not carry SAM-I variant riboswitches (Table S1).

This selective effect of certain polyamines on transcription termination also requires the proper formation of a substructure that is known to be important for ligand binding by SAM-I riboswitch aptamers. Constructs M6 and M7 each carry a single mutation that disrupts the first base pair of the P3 stem (Figure 4A), which is instrumental in forming the ligand binding pocket of SAM-I boswitches.^51^ These mutations are expected to destabilize the P3 stem of the *T. ilyis* variant riboswitch and thus disrupt spermidine-triggered transcription termination. *In vitro* transcription assays reveal that M6 indeed loses all responsiveness to spermidine, whereas the effects of spermidine on M7 are severely reduced (Figure S3B). However, when both disruptive mutations are combined in a single mutant construct (M8), the restored P3 base pair interaction also fully restores spermidine-responsive riboswitch function. Similar outcomes are observed when these mutant constructs are used to control a reporter gene in cells (Figure S3C). Furthermore, spermidine fails to terminate the transcription of a 5′-truncated mutant (M9) (Figure S3D) in which the P1 stem cannot form. This permits formation of the antiterminator structure and a greater proportion of FL transcript production (Figure 4A). These results strongly indicate that the effect of spermidine on transcription termination is due to the specific function of the riboswitch, rather than an effect manifested via polyamine binding to RNA polymerase, the DNA template, or generally to the nascent RNA transcript.

Furthermore, spermidine-dependent transcription termination is specific to the SAM-I variant riboswitch because spermidine does not alter the extent of transcription termination to authentic SAM-I riboswitches tested in this study. Specifically, consensus SAM-I riboswitches from the *T. ilyis* or *Lysinibacillus* sp. BF-4 genome are triggered to strongly terminate transcription by 1 mM SAM, yet remain unaffected by 1 mM spermidine (Figures S2C–S2F). Again, these results are consistent with the hypothesis that the sequence differences between SAM-I and SAM-I variant RNAs result in strict differences in ligand specificity and gene regulation outcomes.

### SAM-I variants function as selective aptamers for spermidine

**Figure 6.**
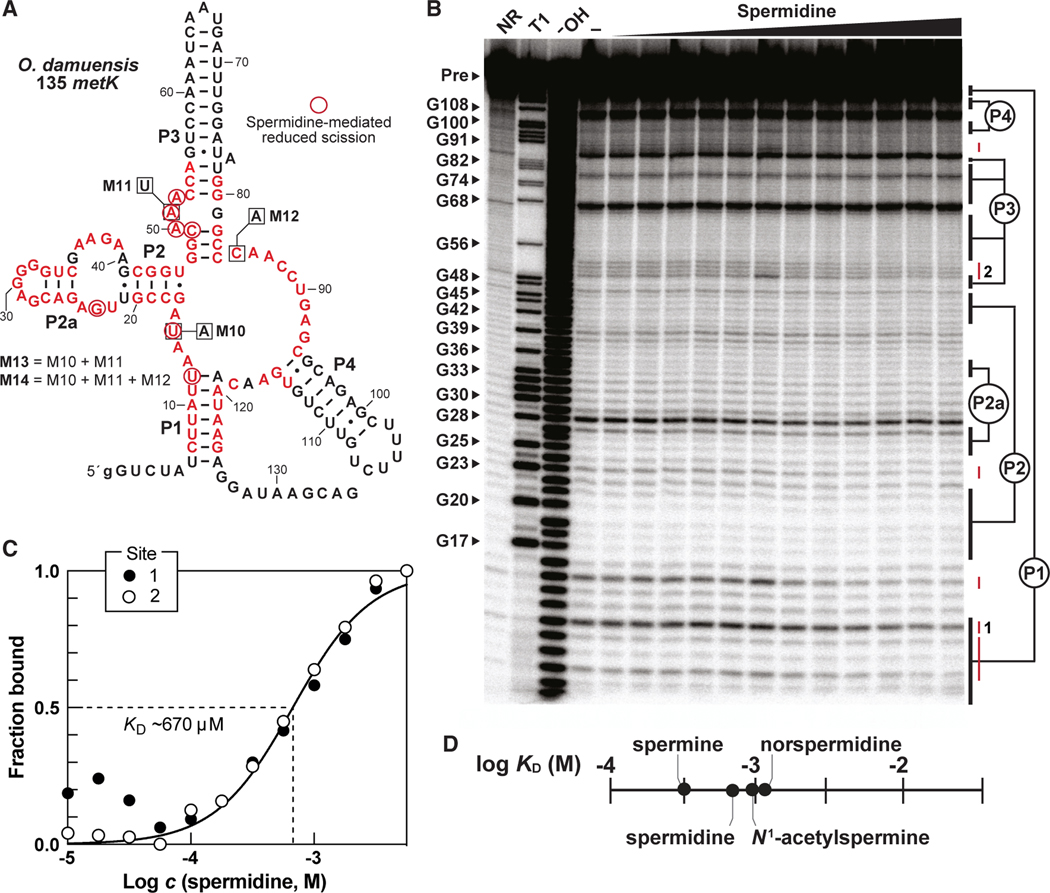
Spermidine modulates the structure of a representative SAM-I variant (A) Sequence and secondary structure model of the 135 *metK* RNA construct derived from the *metK* gene from *O. damuensis*. Lowercase g letter at the 5′ terminus corresponds to a guanosine nucleotide added to the DNA template to enhance production by *in vitro* transcription using T7 RNA polymerase. Mutant constructs M13 and M14 (based on mutations M10, M11, and M12) were used to conduct experiments depicted in Figure S6D. Additional annotations are as described for Figure 3A. (B) PAGE analysis of 5′−^32^P-radiolabeled 135 *metK* RNA subjected to in-line probing without (−) or with a range (10 μM–5.6 mM) of spermidine concentrations representing every quarter log unit. Lanes NR, T1, and ^−^OH indicate RNAs subjected to no reaction, partial digestion with RNase T1 (cleaves after G nucleotides), and partial digestion under alkaline conditions (cleaves after every nucleotide), respectively. The FL RNA precursor (Pre) band and selected bands generated by RNase T1 digestion are indicated. Regions denoted 1 and 2 identify product bands that undergo changes in intensity in response to ligand addition. These sites were used to determine the extent of ligand binding. See Figure S5 for a replicate analysis. (C) Plot of the fraction of RNA bound to the ligand versus the logarithm of the molar concentration (*c*) of spermidine, as estimated by measuring band intensities at sites 1 and 2 as noted in B. K_D_ values were established by determining the concentration of ligand needed to half maximally modulate the RNA structure.^90^ *R*^*2*^ = 0.9663. (D) Plot of the K_D_ values determined for spermidine, spermine, norspermidine, and *N*^1^-acetylspermine. K_D_ values were determined from the plots depicted in (C) and Figures S6A–S6C.

To determine whether SAM-I variant RNAs directly bind spermidine, we used in-line probing^73,74^ assays to assess changes brought about by ligand binding. This method takes advantage of the fact that the rate of spontaneous RNA degradation at each phosphodiester linkage is affected by the local structure of the RNA. Thus, structural changes resulting from ligand binding dictate the pattern of RNA products generated by spontaneous strand scission. A 5′ ^32^P-labeled RNA construct called 135 *metK* encompassing a SAM-I variant aptamer from the *metK* gene of *Oceanobacillus damuensis* (Figure 6A) yielded a pattern of spontaneous RNA cleavage products (Figures 6B and S5) matching the predicted secondary structure (Figure 1B) for this aptamer class. Furthermore, the addition of spermidine triggers modest but quantifiable changes in the pattern of RNA products generated by spontaneous strand scission that are characteristic of selective ligand binding by riboswitch aptamers.

Several observations from the in-line probing data are consistent with the hypothesis that SAM-I variant RNAs function as riboswitches for spermidine. First, most of the locations of nucleotide linkages whose rate of spontaneous cleavage change upon spermidine addition (Figure 6A) correspond to positions that have undergone nucleotide changes relative to the original SAM-I riboswitch consensus (Figure 1). This strongly suggests that nucleotides in the former SAM binding pocket have adapted to recognize a different ligand.

Second, the value for the apparent dissociation constant (K_D_) for spermidine binding by the 135 *metK* RNA construct was measured at ~670 μM (Figure 6C) and ~730 μM (Figure S5), which is similar to the T_50_ value of ~214 μM exhibited for spermidine by the *T. ilyis* riboswitch construct (Figure 4C). Thus, spermidine binding is likely to be responsible for both transcription termination and structural modulation signals observed in these two types of assays. Similar K_D_ values were observed for spermine (~340 μM) and *N*^1^-acetylspermine (~860 μM) (Figures 6D and S6A–S6C), which is consistent with these polyamines functioning like spermidine in promoting transcription termination through the SAM-I variant (Figure 4). Again, the affinity of the RNA for spermine is 2-fold better than for spermidine, which is consistent with the performance of these compounds in transcription termination assays (Figure 4C). Norspermidine also triggers transcription termination (Figure 4) but appears to be bound less tightly (K_D_ > 1 mM) by the variant riboswitch (Figures 6D and S6A–S6C).

Third, the in-line probing data again reveal steep dose-response curves that are indicative of cooperative binding by spermidine (Figures 6C and S6A–S6C). Hill coefficients for spermidine (1.4), spermine (1.4), and *N*^1^-acetylspermine (1.5) were observed. Although rare, other riboswitch classes are also known to bind two or more molecules in a cooperative manner to yield a more “digital” genetic switch that requires smaller changes in ligand concentration to fully modulate gene expression.^6–10^ SAM-I riboswitch aptamers are only known to bind one SAM molecule,^49,51^ whereas the SAM-I variant appears to have altered both its ligand specificity and stoichiometry to cooperatively respond to spermidine and certain other polyamines.

Fourth, nucleotides in the left shoulder of P1, most notably at position 12, undergo reductions in product band intensity (Figure 6B). These nucleotides are predicted to compete with the antiterminator stem as base-pairing partners for nucleotides in the right shoulder of P1 (Figure 4A). Antiterminator stem formation would promote transcription of the FL mRNA and high gene expression. Thus, spermidine-mediated stabilization of the P1 stem, as observed by in-line probing, is consistent with the proposed mechanism of the SAM-I variant riboswitch.

Fifth, the in-line probing effects of spermidine rely on conserved nucleotides as demonstrated by the analysis of mutant versions of the *O. damuensis* 135 *metK* RNA. For example, constructs M13 and M14 (Figure 6A) that carry mutations at positions altered between the SAM-I and SAM-I variant aptamers erode the ability of spermidine to alter the banding pattern observed by in-line probing analysis (Figure S6D). These results again are consistent with the hypothesis that nucleotide changes present in the SAM-I variant alter its ligand specificity from SAM to spermidine, which is reflected by the distinct downstream gene associations (Figure 1C).

## Concluding remarks

Several attempts to identify polyamine-sensing riboswitches have failed to provide satisfying proof that such RNA-sensing devices naturally exist,^43–47^ partly due to the major experimental challenge of addressing the natural nonselective affinity of polyamines for nucleic acids. The present study provides compelling evidence that a riboswitch regulates the expression of polyamine synthesis genes upon selective binding of spermidine. We therefore propose assigning these SAM-I variant RNAs to a distinct spermidine riboswitch class.

Representatives of this spermidine riboswitch class appear (Figure 1B) to adopt the same four-stem architecture as the consensus SAM-I riboswitch,^48–51^ but carry several nucleotide alterations that change the ligand specificity to spermidine. Most other riboswitch classes identified as variants of other more prominent classes generally recognize compounds that are structurally related to the original ligand.^21–25,29,30^ However, some variants of guanidine-I riboswitches^75^ have adapted to recognize strikingly different ligands, including the bacterial signaling nucleotide ppGpp,^26^ the ribose derivative PRPP,^27^ and the nucleotide ADP.^28^ Thus, several precedents exist for riboswitches to repurpose their global architecture via modest mutations to selectively sense fundamentally different ligands. This example of SAM-I riboswitch diversification further highlights the remarkable capacity of natural aptamers to change their ligand specificity to accomplish other regulatory functions that benefit living organisms in their capacity to adapt to changing environments.

Initially, we were intrigued by the fact that the nucleotide changes that distinguish spermidine riboswitch aptamers from the predominant SAM-I class reside at locations forming the SAM binding pocket. Furthermore, the SAM riboswitch positions altered in spermidine riboswitches are responsible for coordinating functional groups in SAM^51^ that are absent from the structurally related MTA compound released upon spermidine production (Figure 2). Thus, MTA initially appeared to be the most plausible ligand for the variant riboswitch that would permit bacteria to halt polyamine biosynthesis, perhaps in response to toxic levels of MTA. However, MTA fails to yield evidence for ligand function in various assays, whereas spermidine exhibits positive effects in all assays. Although defense against MTA toxicity could be achieved by an MTA-sensing riboswitch, this compound should be quickly removed by the methionine salvage pathway.^76^ Perhaps its accumulation is too transient to serve as a reliable indication that spermidine is reaching levels that should trigger a reduction in polyamine biosynthesis.

Spermidine riboswitches are logically associated with genes involved in the direct biosynthesis of spermidine (*speE*, spermidine synthase) and of its immediate precursor putrescine (*speB*, agmatinase). In addition, the riboswitch is also found upstream of the *ldcC* gene (Figure 1C) encoding lysine decarboxylase, which is an enzyme that converts lysine into the polyamine cadaverine (Figure 2). Cadaverine is a precursor of the polyamine aminopropylcadaverine, which is also synthesized through the action of spermidine synthase. Overabundance of these polyamines is potentially detrimental to cells and has been reported to cause decreased protein synthesis and inhibition of cellular growth in both bacterial and eukaryotic species.^77–81^ Our findings suggest that spermidine riboswitches maintain homeostatic levels of polyamines by monitoring the intracellular concentration of spermidine.

The spermidine riboswitch class represents a version of a spermidine-sensing RNA system that exhibits the typical characteristics of a riboswitch, including a selective aptamer domain and a common type of expression platform, and regulates genes directly relevant to the production of polyamines. It is interesting to speculate whether spermidine riboswitches emerged long ago, possibly during the RNA World,^82–84^ a proposed era of life in which genomes and enzymes were solely made of RNA, or if it has recently evolved from a preexisting riboswitch. Given the narrow distribution of the spermidine riboswitch class described in the present study, it seems most likely that this class emerged by mutation of a SAM-I riboswitch to fulfill the cellular need for sensing spermidine and regulating polyamine synthesis. Because of the natural capability of polyamines to bind nucleic acids and affect RNA structure,^85^ we hypothesize that polyamines, like certain metal cations, were among the earliest regulators of RNA structure and activity during the RNA World. It seems certain that additional classes of polyamine riboswitches remain to be discovered.

### Limitations of the study

Spermidine and other polyamines can broadly associate with RNAs to enhance or alter their folding and function, thus making the functional validation of riboswitches sensing and responding to these molecules more problematic. Therefore, a combination of bioinformatic, biochemical, and genetic data were necessary to provide definitive evidence of spermidine riboswitch function. High-resolution structures of the RNA docked to spermidine or its analogs would provide valuable information regarding precisely how SAM-I variant riboswitches have adapted to reject SAM and cooperatively bind multiple polyamine molecules to regulate gene expression, which was not addressed in the present study.

## RESOURCE AVAILABILITY

### Lead contact

Further information and requests for resources should be directed to and will be fulfilled by the communicating author, Dr. Ronald Breaker (ronald.breaker@yale.edu).

### Materials availability

Plasmids and bacterial strains can be obtained by request.

### Data and code availability

- All data used to support the main conclusions of the study are presented in the manuscript or in the supplemental information file.
- This paper does not report original code and the code used has been reported previously.
- No additional data is associated with this paper.

### EXPERIMENTAL MODEL AND STUDY PARTICIPANT DETAILS

#### *Bacillus subtilis* wild-type cells

*Bacillus subtilis* (1A1 strain 168 Δ*trp*) served as the wild-type strain for all experiments. Cells were cultured in rich Luria-Bertani (LB) or glucose minimal media (GMM), supplemented when necessary with 5 μg/mL chloramphenicol, at 37°C under agitation (220 RPM).

#### *Bacillus subtilis* Δ*speD* cells

*Bacillus subtilis* Δ*speD* cells were prepared by genetic knockout using the WT *B. subtilis* (1A1 strain 168 Δ*trp*) as the parent. Cells were cultured in LB or GMM, supplemented when necessary with 5 μg/mL chloramphenicol, at 37°C under agitation (220 RPM).

#### *Bacillus subtilis* Δ*speE* cells

*Bacillus subtilis* Δ*speE* cells were prepared by genetic knockout using the WT *B. subtilis* (1A1 strain 168 Δ*trp*) as the parent. Cells were cultured in LB or GMM media, supplemented when necessary with 5 μg/mL chloramphenicol, at 37°C under agitation (220 RPM).

### METHODS DETAILS

#### Chemicals and biochemicals

All chemicals were purchased from Sigma-Aldrich or Cayman Chemical unless otherwise noted. [α−^32^P]-UTP and [γ−^32^P]-ATP were purchased from PerkinElmer. DNA oligonucleotides (Table S1) were purchased either from Integrated DNA Technologies (IDT), Sigma-Aldrich or the Oligo Synthesis Resource Core at Yale University.

#### Bioinformatics analyses

The automated homology search algorithms CMfinder^86^ and Infernal^87^ were used to identify from the genomic sequences databases RefSeq^52^ versions 80 and 96 sequences similar to SAM-I riboswitches and associated with genes related to polyamine synthesis, as previously described.^18^ The resulting hits were manually examined for sequence differences with the SAM-I riboswitch in positions known to be directly involved in SAM recognition. RNA sequence and secondary structure consensus models and covariation data were manually depicted based on a sequence alignment of 13 representatives of the SAM-I variant.

#### Construction of strains and plasmids

DNA constructs were prepared by GenScript. Each DNA construct carries the *B*. *subtilis lysC* promoter followed by WT or various mutant versions of the SAM-I variant from the *ldcC* gene of *T*. *ilyis*. Each riboswitch region also includes the first eight codons of the downstream ORF (Table S1), which were fused in-frame with the *E. coli lacZ* gene. The constructs were inserted into the *Eco*RI and *Bam*HI sites of a modified version of the pDG1661 vector.^88^ The resulting reporter constructs were then inserted into the *amyE* locus of WT *B. subtilis* (1A1 strain 168 Δ*trp*) or spermidine biosynthesis genes knockout strains (Δ*speD* and Δ*speE*) as noted for each experiment. Reporter constructs insertion was confirmed as previously described.^27^ The knockout strains exhibited growth similar to that of WT cells under the culture conditions use for this study.

#### RNA constructs

Double-stranded DNA templates for *in vitro* transcription carrying a T7 RNA polymerase promoter sequence were generated by PCR using the appropriate primer pairs and synthetic oligonucleotides as templates (Table S1). *In vitro* transcription reactions were performed using 1–2 μg of DNA template and 2 U/μL of T7 RNA polymerase in 80 mM HEPES (pH 7.5 at 20°C), 40 mM DTT, 24 mM MgCl_2_, and 2 mM of each NTP. Reactions were incubated overnight at 37°C, and then treated with TURBO DNase (Thermo Fisher Scientific) for 15 min. Transcription products were then separated by denaturing (8 M urea) polyacrylamide gel electrophoresis (PAGE). RNA products were visualized by UV shadowing, excised, and the resulting gel pieces were crushed and the RNA was eluted in ~400 μL of crush-soak solution (10 mM Tris-HCl [pH 7.5 at 20°C], 500 mM NH_4_OAc, 1 mM EDTA) overnight at 4°C. RNAs were recovered by precipitation with three volumes of cold 100% ethanol followed by centrifugation. Pellets were air-dried, resuspended in deionized water (dH_2_O), and RNA was quantified using a Nanodrop spectrophotometer (Thermo Fisher Scientific). To generate 5′ ^32^P-labeled RNAs for in-line probing, 50 ρmol of RNAs were dephosphorylated using Quick CIP phosphatase (New England Biolabs), and then radiolabeled with 20 μCi [γ−^32^P]-ATP (PerkinElmer) using T4 polynucleotide kinase (New England Biolabs). Radiolabeled RNAs were purified by denaturing (8 M urea) polyacrylamide gel electrophoresis (PAGE) and recovered as described above.

#### In-line probing assays

In-line probing assays were performed as previously described^74^ with some modifications. Briefly, 5′ ^32^P-labeled RNAs were incubated in the presence or absence of different concentrations of ligand candidates at ~20°C for 40–48 h in 50 mM Tris-HCl (pH 7.5 at 20°C), 100 mM KCl, and 2 mM MgCl_2_. The reaction products were separated by denaturing 10% PAGE and visualized using a Typhoon FLA 9500 phosphorimager (GE Healthcare). Dissociation constants were determined by varying the concentration of the ligand added and quantifying the changes in band intensities using ImageJ software (National Institutes of Health) at nucleotide positions exhibiting ligand-induced structural modulation. Values for band intensities were normalized to a non-modulating band, scaled between 0 and 1, and then plotted as a function of the logarithm of the ligand concentration. Apparent *K*_D_ values were calculated using a sigmoidal dose-response equation and GraphPad Prism 9 as previously described.^90^

#### Riboswitch-reporter fusion assays

Analyses of gene expression from riboswitch-reporter fusion constructs were performed by subjecting *B. subtilis* strains carrying reporter constructs to overnight cultures in rich Luria-Bertani medium (LB) (10 g/L tryptone, 5 g/L yeast extract, 10 g/L NaCl) or glucose minimal medium (GMM) (1X Spizizen salts, 0.5% glucose, 0.5 mM CaCl_2_, 2.5 mM MgCl_2_, 5 μM MnCl_2_, 50 μM FeSO_4_, 50 μg/mL tryptophan) supplemented with 5 μg/mL chloramphenicol at 37°C under agitation (220 RPM). Cultures were then diluted 1:100 in LB or 1:10 in GMM media. Cells were grown until late-exponential phase or for 18 h, and β-galactosidase reporter activity was visualized by supplementing the cultures with 50 μg/mL X-gal (Cayman Chemical) or quantified by performing Miller assay as previously described^89^ with some modifications to facilitate *B. subtilis* cells lysis. Briefly, 100 μL of cells were lysed for 20 min in 50 μL of permeabilization buffer (100 mM Tris-HCl [pH 7.8 at 20°C], 32 mM Na_2_HPO_4_, 8 mM DTT, 8 mM cyclohexanediaminetetraacetic acid, 4% Triton X-100) supplemented with 0.75 mg/mL lysozyme and aliquoted in a 96-well microplate. Miller assays were then performed using a Synergy *Neo*2 microplate reader (BioTek) wherein each well contained 50 μL of 4 mg/mL O-nitrophenyl-β-D-galactopyranoside (ONPG) dissolved in Z-buffer (40 mM NaH_2_PO_4,_ 60 mM NaH_2_PO_4_, 1 mM MgSO_4_, 10 mM KCl, 38 mM β-mercaptoethanol). To account for cell densities, β-galactosidase values were adjusted by dividing *V*_max_ by OD_600 nm_.

#### *In vitro* transcription assays

Transcription termination assays were performed as previously described^70^ with some modifications. Briefly, transcription reactions were performed with 100 nM of DNA templates carrying riboswitch constructs whose transcription is driven by the *lysC* promoter sequence from *B. subtilis* (Table S1). Transcription reactions were conducted in 20 mM Tris-HCl (pH 8.0 at 20°C), 100 mM KCl, 2 mM MgCl_2_, 0.01 mg/mL BSA, 1% glycerol, and 0.04 U/μL *E. coli* RNA polymerase holoenzyme (New England Biolabs). Transcription reactions were initiated by the addition of 0.14 mM ApA dinucleotide, 2.5 μM each of ATP and CTP, 1.0 μM UTP, and 2 μCi [α−^32^P]-UTP (PerkinElmer). The mixture was incubated at 37°C for 10 min to allow RNA polymerase to stall at the first G residue of the RNA transcript, which occurs ~16 nts downstream of the transcription start site. Transcription elongation was resumed by the addition of 150 μM each of ATP, CTP and GTP, 50 μM UTP, and 0.1 mg/mL heparin to prevent the RNA polymerase from initiating additional cycles of transcription. The reactions were incubated at 37°C for 30 min. Transcription products were separated by denaturing (8 M urea) 10% PAGE, imaged as described above, and the products were quantified using Image Lab software (Bio-Rad).

### QUANTIFICATION AND STATISTICAL ANALYSIS

Quantification of ^32^P-labeled RNA PAGE band intensities was performed using the ImageJ software for in-line probing assays and Bio-Rad Image Lab software for transcription termination assays. Statistical analysis was performed using GraphPad Prism 9, where *n* represents the number of biological repeats.

## Supplementary Material

1

2

## Figures and Tables

**Table:** KEY RESOURCES TABLE

REAGENT or RESOURCE	SOURCE	IDENTIFIER
Bacterial and virus strains		

*Bacillus subtilis* strain 168 *(trpC2)*	BGSC	1A1
1A1 *speD::kan*	BGSC	BKK29010
1A1 *speE::kan*	BGSC	BKK37500
1A1 *amyE:P_lysC_- TilldcCWT-lacZ cat*	This study	HS240
1A1 *amyE:P_lysC_- TilldcCM1-lacZ cat*	This study	HS241
1A1 *amyE:P_lysC_- TilldcCM2-lacZ cat*	This study	HS243
1A1 *amyE:P_lysC_- TilldcCM3-lacZ cat*	This study	HS244
BKK37500 *amyE:P_lysC_- TilldcCWT-lacZ cat*	BGSC	HS428
BKK29010 *amyE:P_lysC_-TilldcCWT-lacZ cat*	BGSC	HS433
1A1 *amyE:P_lysC_- TilldcCM4-lacZ cat*	This study	HS592
1A1 *amyE:P_lysC_- TilldcCM5-lacZ cat*	This study	HS593
1A1 *amyE:P_lysC_- TilldcCM6-lacZ cat*	This study	HS600
1A1 *amyE:P_lysC_- TilldcCM7-lacZ cat*	This study	HS601
1A1 *amyE:P_lysC_- TilldcCM8-lacZ cat*	This study	HS602
1A1 *amyE:P_lysC_- TilconshypoWT-lacZ cat*	This study	HS603
1A1 *amyE:P_lysC_- TilconshypoM15-lacZ cat*	This study	HS605
BKK37500 1A1 *amyE:P_lysC_- TilconshypoWT-lacZ cat*	This study	HS607
BKK29010 1A1 *amyE:P_lysC_- TilconshypoM15-lacZ cat*	This study	HS608

Chemicals, peptides, and recombinant proteins		

[α−^32^P]UTP	PerkinElmer	Cat #: BLU507H250UC
[γ−^32^P]ATP	PerkinElmer	Cat #: BLU502Z500UC
1,3-Diaminopropane	Sigma-Aldrich	Cat #: D23602–25G
5′-Deoxy-5′-(methylthio)adenosine	Sigma-Aldrich	Cat #: D5011
Agmatine sulfate	Sigma-Aldrich	Cat#: A7127
Bis(3-aminopropyl)amine (nor spermidine)	Sigma-Aldrich	Cat#: I1006
Cadaverine	Sigma-Aldrich	Cat #: C8561
*E. coli* RNA Polymerase, Holoenzyme	New England Biolabs	Cat #: M0551
Ethyl-1,3-diaminopropane	Santa Cruz Biotechnology	Cat: #: sc-331624
L-Arginine	Sigma-Aldrich	Cat #: A5006
L-Ornithine hydrochloride	Sigma-Aldrich	Cat #: O-2375
*N*^1^-acetylspermidine (hydrochloride)	Cayman Chemical	Cat #: 9001535
*N*^1^-acetylspermine (hydrochloride)	Cayman Chemical	Cat#: 17919
*N*-Methyl-1,3-diaminopropane	Sigma-Aldrich	Cat#: 127027–25G
*O*-nitrophenyl-β-D-galactopyranoside (ONPG)	Thermo Fisher Scientific	Cat #: 34055
Propyl-1,3-diaminopropane	Santa Cruz Biotechnology	Cat #: sc-236089
Putrescine	Sigma-Aldrich	Cat #: 51799
Quick CIP	New England Biolabs	Cat #: M0525
RNase T1 (1 U/ml)	Thermo Fisher Scientific	Cat #: AM2283
S-(5′-Adenosyl)-3-thiopropylamine	Sigma-Aldrich	Cat#: 43713
S-(5′-Adenosyl)-L-methionine chloride dihydrochloride	Sigma-Aldrich	Cat #: A7007
Spermine	Sigma-Aldrich	Cat #: 85590
Spermidine trihydrochloride	Sigma-Aldrich	Cat #: S2501
T4 Polynucleotide Kinase	New England Biolabs	Cat #: M0201
TURBO DNase (2 U/ml)	Thermo Fisher Scientific	Cat #: AM2238
X-gal	Cayman Chemical	Cat#: 16495

Deposited data		

*Oceanobacillus damuensis*	RefSeq80 (Ref. #52; https://doi.org/10.1093/nar/gkv11989 )	NZ_LQNF01000010.1/43200–43335
*Bacillus thermoamylovorans*	RefSeq80	NZ_CCRF01000060.1/8653–8516
*Rummeliibacillus stabekisii*	RefSeq80	NZ_CP014806.1/1179153–1179287
*Trichococcus* sp. ES5	RefSeq80	NZ_FJND01000001.1/36715–36568
*Lysinibacillus* sp. BF-4	RefSeq80	NZ_JPUW01000046.1/6598–6731
Environmental DNA	RefSeq80	BMHBC_202880/1413–1562
*Trichococcus ilyis*	RefSeq80	NZ_FJNB01000014.1/83394–83245
*Oceanobacillus massiliensis* str. N’diop	RefSeq80	NZ_HE610978.1/9297–9431
*Trichococcus flocculiformis*	RefSeq80	FJMZ01000015.1/31932–32065
*Viridibacillus arvi*	RefSeq80	NZ_LILB01000001.1/679934–679798
*Sporosarcina* sp. D27	RefSeq80	NZ_AZUC01000044.1/37858–37992
*Ornithinibacillus californiensis*	RefSeq80	NZ_LDUE01000005.1/135850–135988
*Ornithinibacillus contaminans*	RefSeq80	NZ_LDPV02000046.1/73086–73221

Oligonucleotides		

See Table S1 for oligonucleotide sequences.		

Recombinant DNA		

pDG1661tsl	Li et al.^79^	N/A
pDG1661tsl-*TilldcCWT*	GenScript	N/A
pDG1661tsl-*TilldcCM1*	GenScript	N/A
pDG1661tsl-*TilldcCM2*	GenScript	N/A
pDG1661tsl- *TilldcCM3*	GenScript	N/A
pDG1661tsl-*TilldcCM4*	GenScript	N/A
pDG1661tsl-*TilldcCM5*	GenScript	N/A
pDG1661tsl-*TilldcCM6*	GenScript	N/A
pDG1661tsl-*TilldcCM7*	GenScript	N/A
pDG1661tsl-*TilldcCM8*	GenScript	N/A
pDG1661tsl*-TilconshypoWT*	GenScript	N/A
pDG1661tsl*-TilconshypoM15*	GenScript	N/A

Software and algorithms		

ImageJ	NIH	https://imagej.nih.gov/ij/
Image Lab	Bio-Rad	https://www.bio-rad.com/en-us/product/image-lab-software?ID=KRE6P5E8Z
Prism 9	GraphPad	https://www.graphpad.com/

## Highlights

- Natural spermidine-sensing riboswitches exist in bacteria
- Rare SAM-I riboswitch variants have adapted to sense spermidine
- Spermidine riboswitches repress polyamine biosynthesis genes
