# Supplementary material for: A spermidine riboswitch class in bacteria exploits a close variant of an aptamer for the enzyme cofactor S-adenosylmethionine
Source: Cell Rep. Author manuscript; Available in PMC 2024 Feb 9. (PMC10853860; doi:10.1016/j.celrep.2023.113571)
Supplement: 1 [file NIHMS1954802-supplement-1.pdf]

Cell Reports, Volume *42*

Supplemental information

A spermidine riboswitch class in bacteria exploits a close variant of an aptamer for the enzyme cofactor *S*-adenosylmethionine

Hubert Salvail, Aparaajita Balaji, Adam Roth, and Ronald R. Breaker

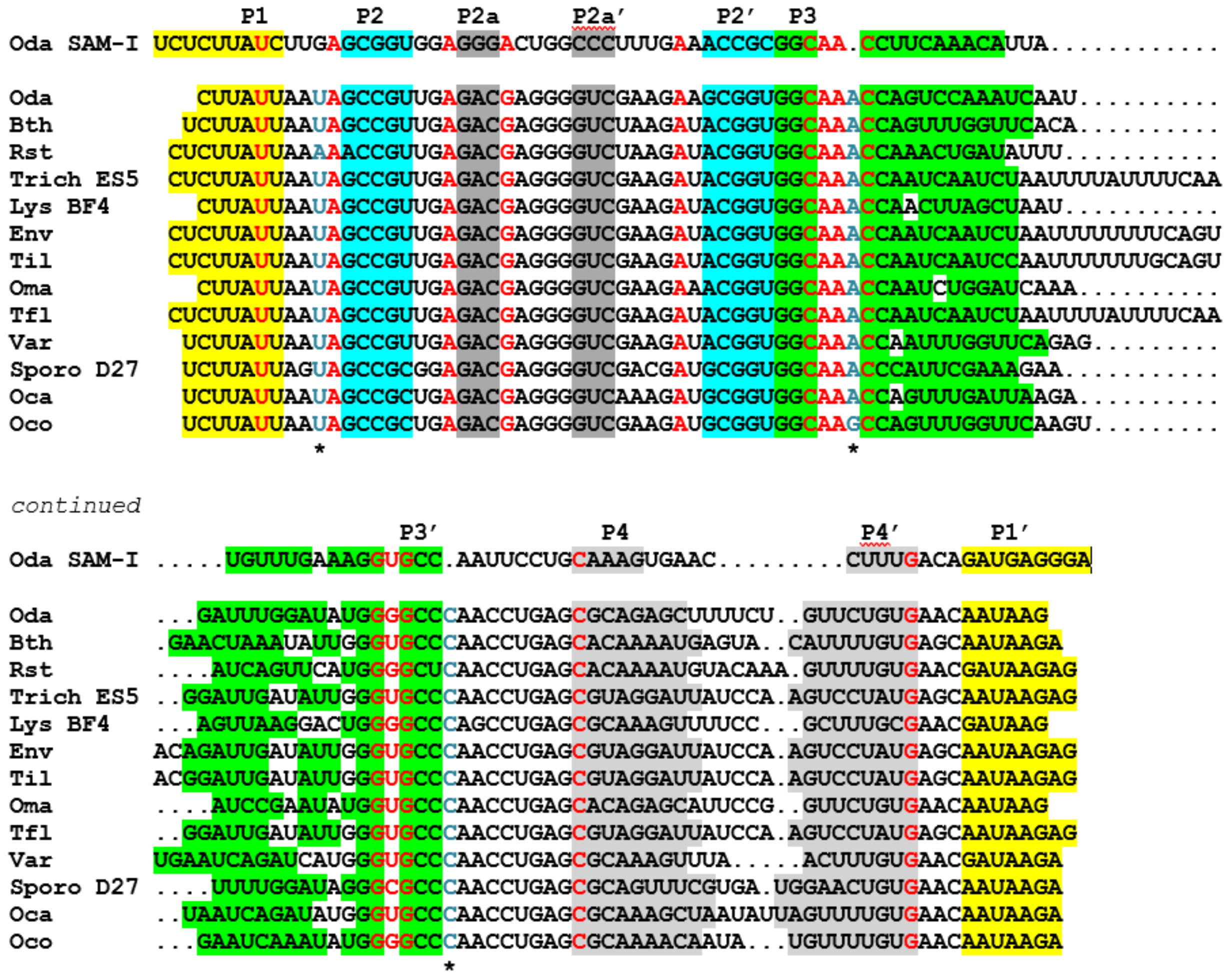

Figure S1, Related to Figure 1. Sequence alignment of the known SAM-I variant RNAs associated with spermidine biosynthesis genes

Top sequence: A representative SAM-I riboswitch sequence from *Oceanobacillus damuensis* (Oda SAM-I). Remaining sequences: Thirteen SAM-I variant RNAs from *Oceanobacillus damuensis* (Oda), *Bacillus thermoamylovorans* (Bth), *Rummeliibacillus stabekisii* (Rst), *Trichococcus* sp. ES5 (Trich ES5), *Lysinibacillus* sp. BF-4 (Lys BF4), environmental DNA (Env), *Trichococcus ilyis* (Til), *Oceanobacillus massiliensis* str. N'diop (Oma), *Trichococcus flocculiformis* (Tfl), *Viridibacillus arvi* (Var), *Sporosarcina* sp. D27 (Sporo D27), *Ornithinibacillus californiensis* (Oca), and *Ornithinibacillus contaminans* (Oco). Predicted base-paired structures P1 through P4 are designated with colored shading. Red letters correspond to nucleotides whose identity is conserved in more than 97% of the representatives of SAM-I riboswitch aptamers. Blue letters (see asterisks) designate notable changes that distinguish SAM-I variant RNAs from the predominant SAM-I class.

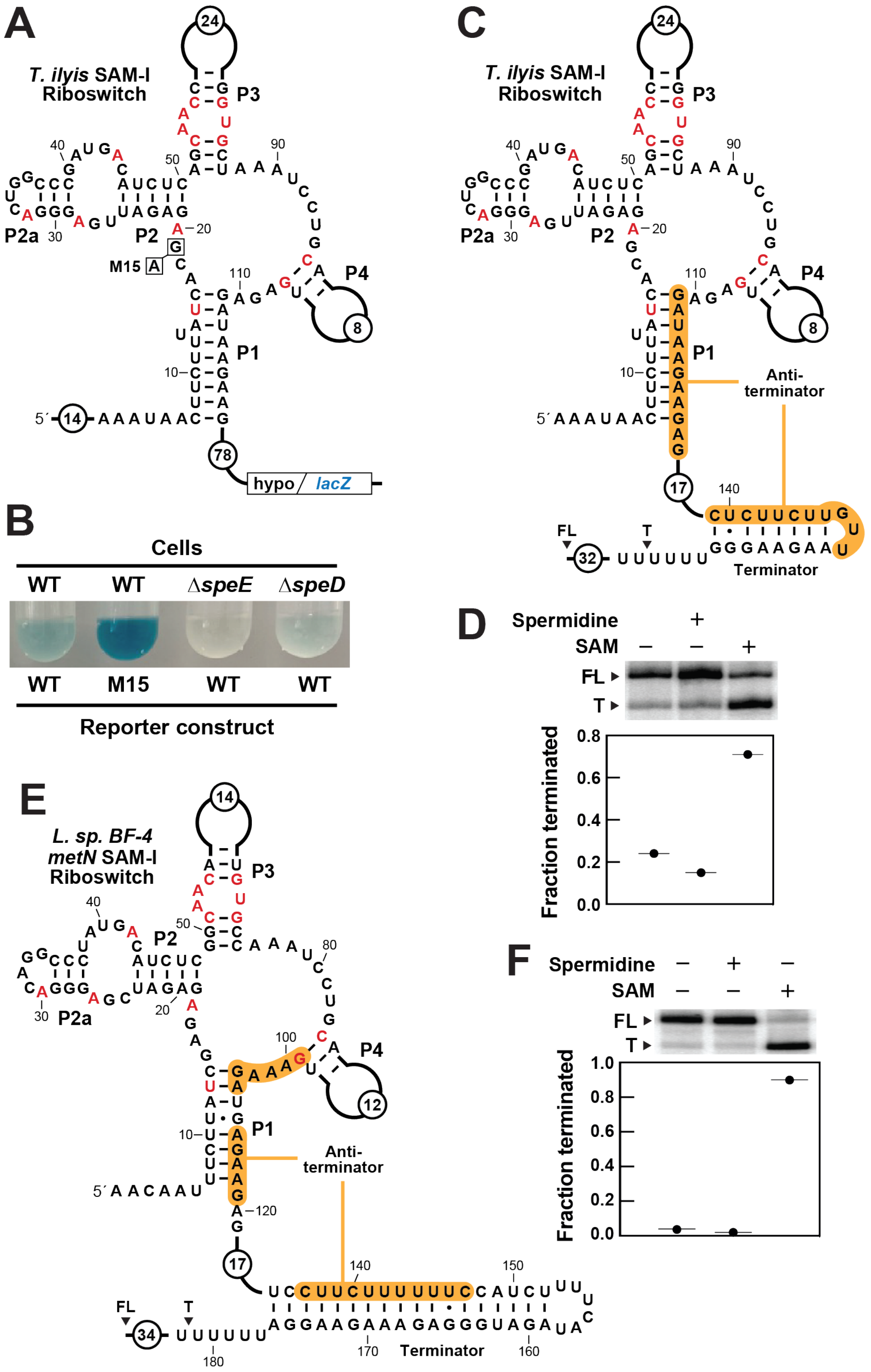
A
T. ilyis SAM-I
Riboswitch
P3
P2a
P2
M15
P4
P1
hypo
lacZ
B
Cells
WT
WT
ΔspeE
ΔspeD
WT
M15
WT
WT
Reporter construct
C
T. ilyis SAM-I
Riboswitch
P3
P2a
P2
P4
P1
Anti-
terminator
FL
T
Terminator
D
Spermidine
SAM
FL
T
Fraction terminated
0.8
0.6
0.4
0.2
0.0
E
L. sp. BF-4
metN SAM-I
Riboswitch
P3
P2
P2a
P4
P1
Anti-
terminator
FL
T
Terminator
F
Spermidine
SAM
FL
T
Fraction terminated
1.0
0.8
0.6
0.4
0.2
0.0

Figure S2, Related to Figure 3C and Figure 4. SAM-I riboswitch representatives do not respond to genetic disruptions in spermidine biosynthesis and do not promote spermidine-mediated transcription termination

(A) Sequence and secondary structure model for a SAM-I riboswitch-reporter translational fusion construct. The *T. ilyis* SAM-I riboswitch associated with a gene encoding for a hypothetical protein (hypo) was fused to the *E. coli lacZ* gene. Nucleotides in red correspond to highly conserved nucleotides from the SAM-I consensus model (**Figure 1A**). The boxed nucleotide identifies the construct representing mutant M15 used in the experiment depicted in B.

(B) Effect of spermidine biosynthesis genes mutations on a SAM-I riboswitch-reporter construct. *B. subtilis* WT, Δ*speD*, and Δ*speE* strains carrying either the WT or M15 riboswitch-reporter fusion constructs were grown for 18 h in GMM medium. Cultures were then supplemented with X-gal to visualize the reporter activity. Data are representative of two independent experiments, which gave similar results.

(C) Sequence and secondary structure model of a SAM-I riboswitch associated with a gene annotated as "hypothetical" (hypo) from *T. ilyis*. Encircled numbers indicate nucleotides not shown. Red letters correspond to highly conserved nucleotides as defined in the SAM-I consensus model (**Figure 1A**). The approximate 3′ end of terminated (T) transcripts and the 3′ end of full-length (FL) transcripts are indicated. FL carries an additional 32 nucleotides (encircled number) not shown.

(D) Top: PAGE analysis of single-round transcription termination assays performed with the *T. ilyis* SAM-I construct in the absence of ligand (–), or in the presence of 1 mM spermidine or SAM. Bottom: Values for the fraction of terminated RNA transcripts derived from the PAGE gel.

(E) Sequence and secondary structure model of a SAM-I riboswitch associated with the *metN* gene from *L. sp. BF-4*. Annotations are as described for C.

(F) Transcription termination analyses of the *L. sp. BF-4* SAM-I riboswitch. Annotations are as described for D.

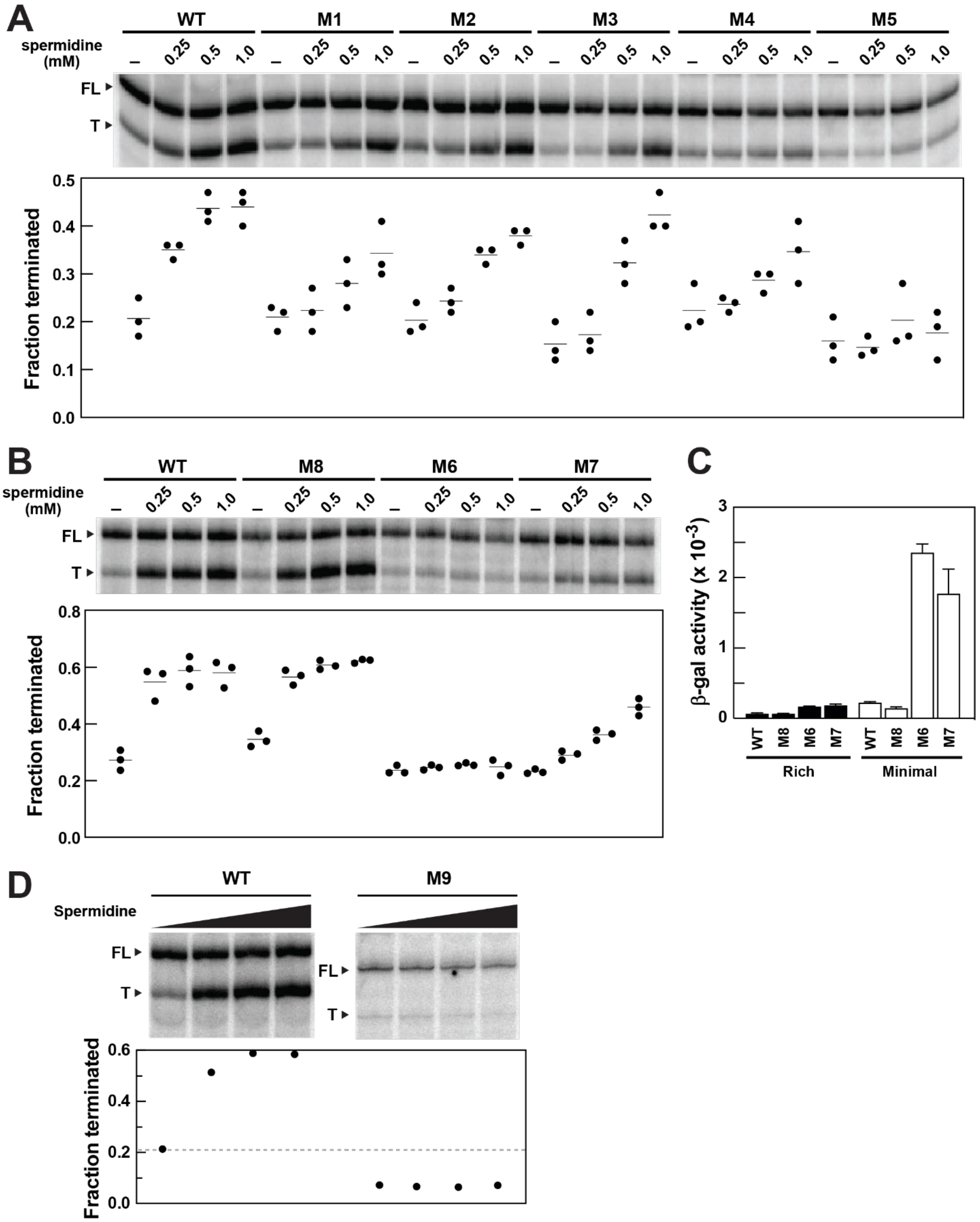
A
WT
M1
M2
M3
M4
M5
spermidine (mM)
–
0.25
0.5
1.0
FL
T
Fraction terminated
0.0
0.1
0.2
0.3
0.4
0.5
B
WT
M8
M6
M7
0.6
0.8
C
β-gal activity (x 10^-3)
Rich
Minimal
D
M9
Spermidine

Figure S3, Related to Figure 4. Transcription termination analyses to evaluate the effects of mutations on a SAM-I variant riboswitch

(A) Top: PAGE analysis of single-round transcription termination assays of spermidine effect on WT and mutant versions of the *T. ilyis ldcC* SAM-I variant construct (**Figure 4A**). Bands corresponding to terminated (T) and full-length (FL) RNA transcripts are indicated. Bottom: Values for the fraction of terminated (T) RNA transcripts derived from three independent experiments ($n = 3$).

Notes: Constructs M1, M2, and M3 each carry a single mutation at a nucleotide position that is distinct between the SAM-I and SAM-I variant RNAs, and all three exhibit reduced spermidine responsiveness compared to the WT construct. Mutants M4 and M5 combine two (M1 and M2) or three (M1, M2 and M3) mutations that further disrupt the effects of spermidine on transcription termination.

(B) Top: PAGE analysis of representative single-round transcription termination assays evaluating the effects of spermidine on WT and mutant constructs M6, M7, and M8 of the *T. ilyis ldcC* SAM-I variant riboswitch (**Figure 3A**). Bands corresponding to terminated (T) and full length (FL) RNA transcripts are designated. Bottom: Values for the fraction of terminated RNA transcripts derived from three independent experiments. Each cluster of three datapoints corresponds to the construct and reaction conditions noted for the gel depicted immediately above.

(C) β-galactosidase assay results generated with WT *B. subtilis* cells carrying either WT or mutant M6, M7, or M8 versions of the *T. ilyis ldcC* SAM-I variant riboswitch-reporter constructs (**Figure 3A**). Cells were grown in LB or GMM media in late exponential phase and reporter activity was then quantified. Error bars indicate the standard deviation for activity values derived from three independent experiments (n = 3).

(D) Top: PAGE analysis of single-round transcription termination assays evaluating the effects of increasing spermidine concentrations (0, 0.25, 0.5 and 1 mM) on WT and the 5′-truncated M9 construct of the *T. ilyis ldcC* SAM-I variant riboswitch. Bands corresponding to terminated (T) and full length (FL) RNA transcripts are designated. Bottom: Plot of the values for the fraction of terminated RNA transcripts derived from the PAGE gel depicted.

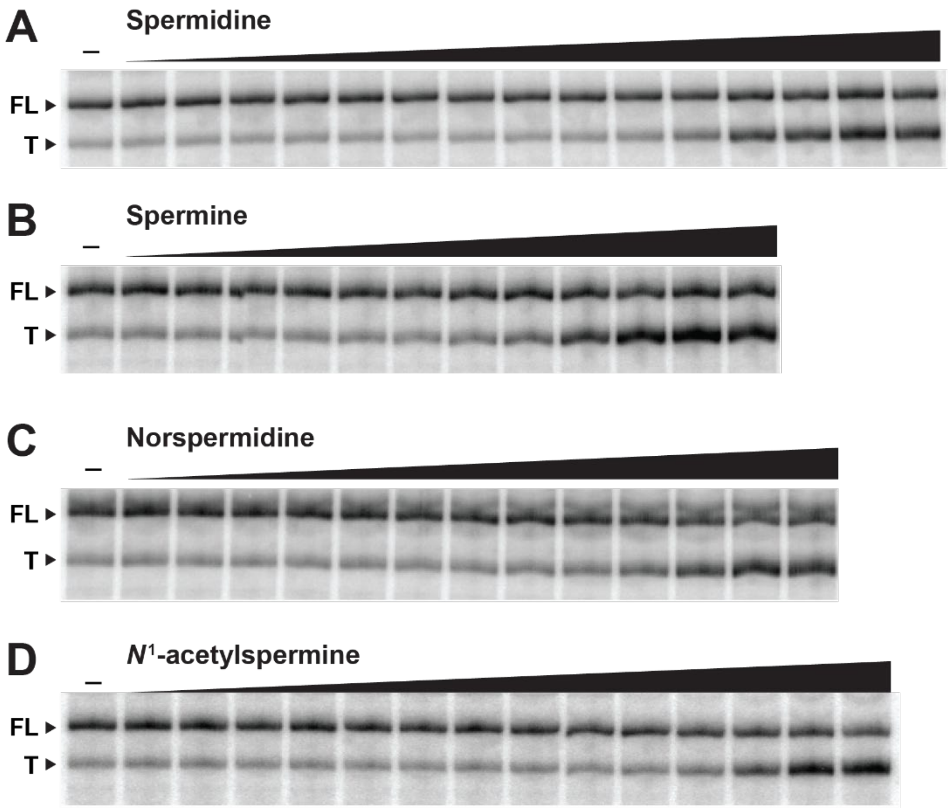

Figure S4, Related to Figure 4. Transcription termination analysis to establish the T_{50} values for spermidine analogs

(A) PAGE analysis of single-round transcription termination assays of *T. ilyis ldcC* SAM-I variant construct without (–), or with a range (100 nM to 4.7 mM) of spermidine concentrations (3rd log increments). Terminated (T) and full-length (FL) transcripts are as indicated.

(B) Analysis as described in A for spermine (100 nM to 468 μM).

(C) Analysis as described in A for norspermidine (100 nM to 1 mM).

(D) Analysis as described in A for N^1-acetylspermine (100 nM to 2.14 mM).

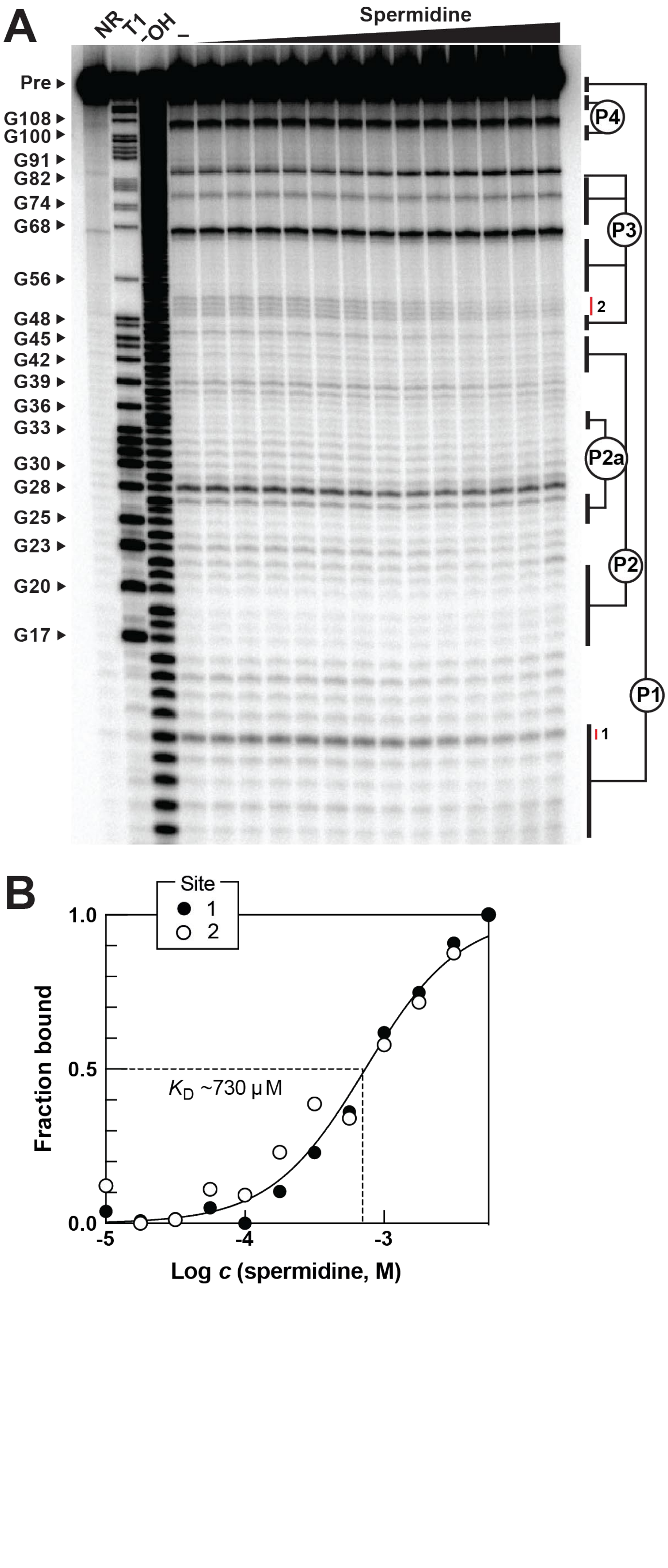
A
NR
T1
-OH
–
Spermidine
Pre
G108
G100
G91
G82
G74
G68
G56
G48
G45
G42
G39
G36
G33
G30
G28
G25
G23
G20
G17
P4
P3
2
P2a
P2
P1
1
B
Site
1
2
1.0
0.5
0.0
Fraction bound
K_D ~730 μM
-5
-4
-3
Log c (spermidine, M)

Figure S5, Related to Figure 6B. Replicate in-line probing assay with the 135 *metK* RNA.

(A) Analysis of 5′-^{32}P-radiolabeled 135 *metK* RNA subjected to in-line probing without (–), or with a range (10 µM to 5.6 mM) of spermidine concentrations representing every quarter log unit. Annotations are as described for **Figure 6B**.

(B) Plot of the fraction of RNA bound to ligand versus the logarithm of the molar concentration (*c*) of spermidine, as estimated by measuring band intensities at sites 1 and 2 as denoted in B. $R^2 = 0.9843$.

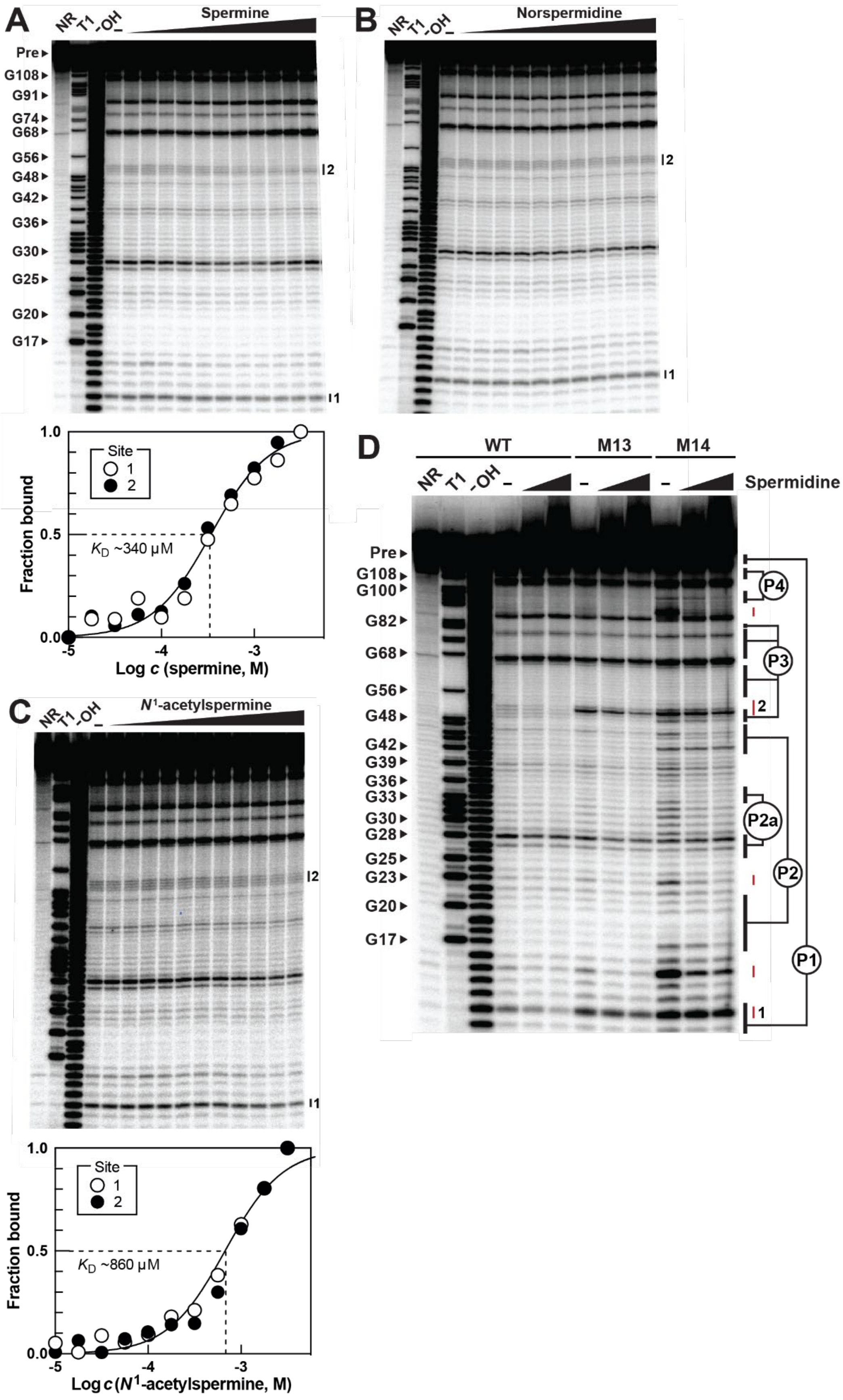
A
NR
T1
-OH
Spermine
Pre
G108
G91
G74
G68
G56
G48
G42
G36
G30
G25
G20
G17
2
1
B
Norspermidine
Site
Fraction bound
K_D ~340 µM
Log c (spermine, M)
C
N1-acetylspermine
K_D ~860 µM
Log c (N1-acetylspermine, M)
D
WT
M13
M14
Spermidine
G100
G82
G39
G33
G28
G23
P4
P3
P2a
P2
P1

Figure S6, Related to Figure 6. In-line probing analysis to determine the apparent K_D values for spermidine analogs and the effect on spermidine binding of mutations to nucleotides that distinguish SAM-I variant riboswitch aptamers from the SAM-I consensus

(A) Top: 5′-^{32}P-radiolabeled 135 *metK* RNA was subjected to in-line probing without (–) or with a range (10 μM to 10 mM) of spermine concentrations representing every quarter log. Additional annotations are as described for **Figure 6B**. Bottom: Plot of the fraction of 135 *metK* RNA bound to the ligand versus the logarithm of the molar concentration (*c*) of spermine. Additional annotations are as described for **Figure 6C**.

(B) Analysis as described in A for norspermidine. Note that the band intensity changes for norspermidine at sites 1 and 2 were considered insufficient to accurately determine a K_D value, but we estimate that the value is greater than 1 mM.

(C) Analysis as described in A for N^1-acetylspermine (10 μM to 3.2 mM).

(D) PAGE analysis of 5′-^{32}P-radiolabeled WT, M13, and M14 constructs of the *O. damuensis* 135 *metK* RNA (**Figure 6A**) subjected to in-line probing without (–) or with 1 mM or 5.6 mM spermidine. Additional annotations are as described for **Figure 6B**.
